# Genome-Wide Identification of the VQ Protein Gene Family of Tobacco (*Nicotiana tabacum* L.) and Analysis of Its Expression in Response to Phytohormones and Abiotic and Biotic Stresses

**DOI:** 10.3390/genes11030284

**Published:** 2020-03-07

**Authors:** Cuihua Liu, Hai Liu, Changyong Zhou, Michael P. Timko

**Affiliations:** 1 Citrus Research Institute, Southwest University, Chongqing 400712, China; lch123@email.swu.edu.cn; 2 Department of Biology, University of Virginia, Charlottesville, VA 22903, USA; hl9h@virginia.edu

**Keywords:** VQ protein, tobacco, phytohormone, abiotic stress, pathogen infection

## Abstract

VQ motif-containing proteins (VQ proteins) are transcriptional regulators that work independently or in combination with other transcription factors (TFs) to control plant growth and development and responses to biotic and abiotic stresses. VQ proteins contain a conserved FxxhVQxhTG amino acid motif that is the main element of its interaction with WRKY TFs. We identified 59 members of the tobacco (*Nicotiana tabacum* L.) *NtVQ* gene family by in silico analysis and examined their differential expression in response to phytohormonal treatments and following exposure to biotic and abiotic stressors. NtVQ proteins clustered into eight groups based upon their amino acid sequence and presence of various conserved domains. Groups II, IV, V, VI, and VIII contained the largest proportion of *NtVQ* gene family members differentially expressed in response to one or more phytohormone, and NtVQ proteins with similar domain structures had similar patterns of response to different phytohormones. *NtVQ* genes differentially expressed in response to temperature alterations and mechanical wounding were also identified. Over half of the *NtVQ* genes were significantly induced in response to *Ralstonia solanacearum* infection. This first comprehensive characterization of the *NtVQ* genes in tobacco lays the foundation for further studies of the *NtVQ*-mediated regulatory network in plant growth, developmental, and stress-related processes.

## 1. Introduction

Tobacco (*Nicotiana tabacum* L.) is an agriculturally important crop in the family Solanaceae and is one of the most studied model plant systems [1,2]. Despite a downturn in tobacco production for human consumption, it is still widely used in basic and applied plant research because it can be easily maintained and manipulated in cell culture, readily genetically transformed and regenerated, and can be easily grown in the field and greenhouse. There are also high-quality draft genomes for both cultivated tobacco varieties and wild progenitor species *N. sylvestris* and *N. tomentosiformis*, and a large number of transcriptomic datasets providing information on gene expression characteristics for cultivated tobacco in comparison to its wild relatives and other members of the Solanaceae [3].

Like all plants, tobacco must be able to withstand a wide array of abiotic and biotic stressors and therefore has evolved elaborate mechanisms to respond to various external stimuli [4]. Such responses are governed by a sophisticated regulatory network involving endogenously and exogenously formed signals in the form phytohormones and other compounds that activate signaling pathways that modulate transcriptional and post-transcriptional processes [5,6,7,8,9]. Understanding the complexity of transcription factors (TFs) and transcriptionally active proteins (TAPs, syn. transcription associated proteins) present in the genome is a necessary step to understand the broad aspects of how growth and development and response to environmental cues and stressors occurs.

Among the major transcriptional regulators found in plants are the VQ motif-containing proteins (designated as VQ proteins) that work independently or in combination with other TFs to regulate diverse plant growth and developmental processes and responses to biotic and abiotic stresses [10]. The VQ proteins, identified by the presence of a conserved FxxhVQxhTG amino acid motif, have been found in many plant species including both monocotyledons and dicotyledons [11,12,13,14,15,16,17,18,19,20,21,22,23,24,25]. These proteins are further classified into several types based on the terminal three amino acids in the conserved motifs. For example, there are six different types of motifs in Arabidopsis (e.g., LTG, LTS, LTD, FTG, VTG and YTG) [11]. Although initially thought to be a plant-specific transcriptional regulator, VQ proteins have been recently identified in some fungi, lower animals, and bacteria, suggesting that this gene family has an ancient origin [26].

VQ proteins have been reported to play crucial regulatory roles in vegetative plant growth and differentiation, seed development, and biotic or abiotic stress responses. For example, the mitogen-activated protein (MAP) kinase 3/6-targeted VQ protein 1 (MVQ1, also known as AtVQ4) plays a negative role in regulating pathogen-associated molecular pattern (PAMP)-induced resistance to pathogens [27]; AtVQ9 negatively regulates salinity stress responses [28]; HAIKU1 (IKU1, also known as AtVQ14) exhibits an essential role in the determination of Arabidopsis seed size through the IKU pathway [29]; AtCaMBP25/AtVQ15 functions as a negative effector of osmotic stress tolerance during seed germination and seedling growth [30]; AtVQ20 acts as a key player to modulate pollen development, germination, and tube growth [31]; MKS1/AtVQ21 contributes to the MAP kinase 4 (MPK4)-activated pathogen defense and the overexpression of *AtVQ21/MKS1* resulted in increased tolerance to *Pseudomonas syringae* pv. tomato in transgenic *Petunia* plants [32,33,34]; jasmonate-associated VQ motif protein 1 (JAV1, also known as AtVQ22) acts as a repressor protein in JA-mediated defense responses against necrotrophic pathogens and herbivorous insects without influencing plant growth and development [35,36]; SIB1/AtVQ23 and SIB2/AtVQ16 function as activators in plant defense against necrotrophic pathogens [37]. Additionally, AtVQ18 and AtVQ26 were found to fine-tune seed germination and seedling establishment [38], while AtVQ29 was reported to interact with Phytochrome-Interacting Factor 1 (PIF1), leading to the repression of seedling de-etiolation [39]. The banana fruit VQ5 acts as a repressor of cold-responsive transcription factor MaWRKY26 involved in activating JA biosynthesis [25].

Despite its biological and economic significance, little information is currently available on the *VQ* protein gene family in cultivated tobacco (*N. tabacum* L.) and the role(s) of these transcriptional regulators in controlling plant growth and development and response to various biotic and abiotic stressors. To address this lack of information we conducted a comprehensive in silico analysis of available tobacco genomic sequence data and identified 59 members of the VQ protein gene family. We further examined the structure of various *NtVQ* genes and their encoded proteins, determined their phylogenetic relationship, and defined conserved motifs within the different *NtVQ* family members. To better understand their potential functional roles within the tobacco plant, we carried out gene expression analysis in response to treatment with various phytohormone, and biotic and abiotic stress factors. We also correlated differential gene expression data to the presence of conserved *cis*-regulatory elements in the promoters of the *NtVQ* genes characterized in this study. Our findings provide a foundation for subsequent follow-up studies to define the nature of the interaction of *NtVQ* proteins with other important TFs and TAPs.

## 2. Materials and Methods

### 2.1. Plant Materials, Growth Conditions and Stress Treatments

Tobacco (*Nicotiana tabacum* L var. K326) seeds were collected from plants grown in the greenhouse at the University of Virginia. The seeds were surface sterilized by treating them in 75% (v/v) ethanol for 30 s, followed by 5 min in 20% (v/v) Clorox^®^ bleach (Clorox, Oakland, CA, USA) and 5 min in 20% (v/v) MetriCide™ 28 (Metrex Research, Orange, CA, USA). After surface sterilization, the seeds were germinated on agar plates containing MS basic media [40]. After germination, the seedlings were transferred to a new MS medium plate and grown in a controlled temperature room maintained at 23–25 °C under a 12 h light/12 h dark light cycle and allowed to grow till they were two weeks old. The seedlings were then used for the various treatments described below. Two-week-old seedlings to be used for pathogen treatment were transferred to peat pellets and grown for two additional weeks under the same light and temperature conditions described above. Plants were watered on a weekly basis.

For phytohormonal treatment, two-week-old seedlings were sprayed with the respective phytohormonal solutions. Seedlings were harvested at 0 h, 3 h, and 24 h post treatments. Six individual seedlings were harvested at each time point for each treatment in this experiment. To reduce interplant variation, six individual seedlings were pooled and treated as a single sample for RNA extraction and gene expression analysis.

For phytohormone response analysis the following treatments were used: 50 μM 2,4-dichlorophenoxyacetic acid (2,4-D); 2 mM salicylic acid (SA); 100 μM methyl jasmonate (MeJA); 1 mM ethephon (ETH); and 100 μM abscisic acid (ABA). To prepare 2,4-D solutions, the powder was dissolved in sterile water; the other phytohormones were prepared with Dimethyl Sulfoxide (DMSO) as 1000x stock. The control plants for phytohormone treatments with SA, JA (MeJA), ETH, and ABA were sprayed with 0.1% DMSO (v/v) in sterile water, whereas the control plants for 2,4-D treatment were sprayed with sterile distilled water.

For abiotic-stress treatments, two-week-old tobacco seedlings were placed in a growth chamber at 4 °C and 35 °C, respectively, for 0 h, 3 h, and 24 h. For mechanical wounding treatment, leaves of two-week-old seedlings were squeezed with a pair of tweezers twice. As described above, six individual seedlings were collected at 0 h, 3 h, and 24 h and pooled for each sample. Control plants were grown under standard light and temperature as described above.

For the pathogen treatment, *Ralstonia solanacearum* Y45 (OD = 0.001), isolated from Yunnan, China, was grown in liquid TTC media to saturation and 50 μL of the pathogenic slurry was inoculated onto leaves of four-week-old tobacco plants using a 1 mL syringe. For control treatment, plants were inoculated with 10 mM MgCl_2_. The treated plants were grown in a growth chamber maintained at 30 °C with a relative humidity of 80% and a 12 h light/12 h dark cycle. Leaves from three infected plants were collected at 0, 1, and 3 days after treatment and pooled per replicated sample.

### 2.2. Identification of VQ Genes in Tobacco and Gene Structure Analysis

The *NtVQ* family members were predicted based on the conserved VQ motif using HMMER software (v 3.1b2, http://hmmer.org/). Briefly, a profile hidden Markov model (HMM) was built based on the seed alignment of all VQ sequences retrieved form the Pfam database (Pfam 30.0, https://pfam.xfam.org/). This VQ HMM was then used to search against *N. tabacum* protein database downloaded from National Center for Biotechnology Information (NCBI, https://www.ncbi.nlm.nih.gov/assembly/GCF_000715135.1).

Coding sequences (CDS) of all the *NtVQ* genes were aligned with their corresponding genomic DNA sequences to determine the intron and exon structures. The number of amino acids (aa), open reading frame (ORF) length, molecular weight (MW), isoelectric point (pI), the grand average of hydropathicity (GRAVY), instability index and aliphatic index for each gene were obtained using ExPASy (http://web.expasy.org/protparam/). The WoLF PSORT prediction tool (https://www.genscript.com/wolf-psort.html) was used to analyze the subcellular location of 59 *NtVQs*.

### 2.3. Multiple Alignment and Phylogenetic Analysis

Thirty-four (34) Arabidopsis VQ proteins sequences (*Arabidopsis thaliana* TAIR10) and 26 tomato VQ protein sequences were downloaded from the Phytozome database (https://phytozome.jgi.doe.gov/pz/portal.html). Phylogenetic relationships among gene family members were inferred using the Maximum Likelihood method based on the JTT matrix-based model [41]. The tree with the highest log likelihood (−2031.66) was known. The percentage of trees in which the associated taxa clustered together was shown next to the branches. Initial tree(s) for the heuristic search were obtained automatically by applying Neighbor joining and BioNJ algorithms to a matrix of pairwise distances estimated using a JTT model, and then selecting the topology with superior log likelihood value. The tree was drawn to scale, with branch lengths measured in the number of substitutions per site. There was a total of 35 positions in the final dataset. Evolutionary analyses were conducted in MEGA 7.0 [42]. The alignments of full-length sequences of NtVQ proteins were aligned using BioEdit software (http://www.mbio.ncsu.edu/BioEdit/bioedit) for their types of conserved VQ motif analysis.

To evaluate structural divergence, the 59 NtVQ proteins were examined using the Multiple Expectation Maximization for Motif Elicitation (MEME) online program (http://meme-suite./org/tools/meme) with the following parameters: number of repetition = any; maximum number of motifs = 20 [43,44].

### 2.4. Gene Duplication and Synonymous (Ks) and Nonsynonymous (Ka) Substitution Calculation

The analysis of paralogous genes among *NtVQ* family was found using the Clustal Omega online software (https://www.ebi.ac.uk/Tools/msa/clustalo/). The chosen pairs were confirmed by comparing the positions in the phylogenetic tree and conserved motifs from MEME analysis. Orthologous VQ proteins between tobacco and Arabidopsis were defined by BLASTP search in NCBI database at a 1e-5 significance level. The ones with the best score were chosen to be the orthologous pairs. The Ka and Ks were calculated to assess the selection history and divergence time of gene families. The number of synonymous (Ks) and nonsynonymous (Ka) substitutions of duplicated VQ genes was computed by using the KaKs_calculator 2.0 with the MYN method [45]. The divergence time (T) was calculated using the formula T = Ks/(2 × 6.1 × 10^−9^) × 10^−6^ million years ago (MYA) with a mutation rate of 1 × 10^−8^ [46,47].

### 2.5. Promoter Analysis

Genomic DNA sequences of ~2 kb upstream of the start codon “ATG” of each predicted *NtVQ* were downloaded from the NCBI database and screened for *cis*-acting elements using PlantCare (http://bioinformatics.psb.ugent.be/webtools/plantcate/html) [48].

### 2.6. RNA Extraction and Quantitative Reverse-Transcription Polymerase Chain Reaction (qRT-PCR) Analysis

Total RNA was extracted from tobacco whole seedlings using TRIZOL (ThermoFisher Scientific, Waltham, MA, USA) according to the manufacturer’s instructions. First-strand cDNA synthesis was accomplished using QuantiTect Reverse Transcription Kit (Qiagen, Hilden, Germany) based on the manufacturer’s instructions. NCBI Primer-blast was used to design primers specific to each *NtVQ* gene (Supplementary Table S1), and tobacco *Elongation Factor 1-α* (*NtEF1α*) was used as internal control [49]. qRT-PCR was performed in a 10 μL volume containing 5 μL of 2 × iTaq^TM^ Universal SYBR Green Supermix (Bio-Rad Laboratories, Hercules, CA, USA), 0.5 μL diluted cDNA template, 0.25 μL of each specific primer, and 4 μL ddH_2_O. PCR reaction conditions were as follows: 95 °C for 30 s, followed by 40 cycles of denaturation at 95 °C for 5 s, annealing and extension at 60 °C for 40 s. The relative expression level of each gene was calculated by 2^−^^△△Ct^ method compared with that of the control sample at 0 h. Statistical analysis was conducted using the STATPAK tool (http://statpages.info/anova1sm.html), which performs both a one-way ANOVA and the Tukey HSD (“Honestly Significant Difference”) post hoc test to calculate the significant difference of treated samples among different time points compared to the control samples. The CK calibrator refers to the gene expression level of the control plant at 0 h.

## 3. Results

### 3.1. Identification of VQ Family Members and Sequence Analysis in Tobacco

**Figure 1:**
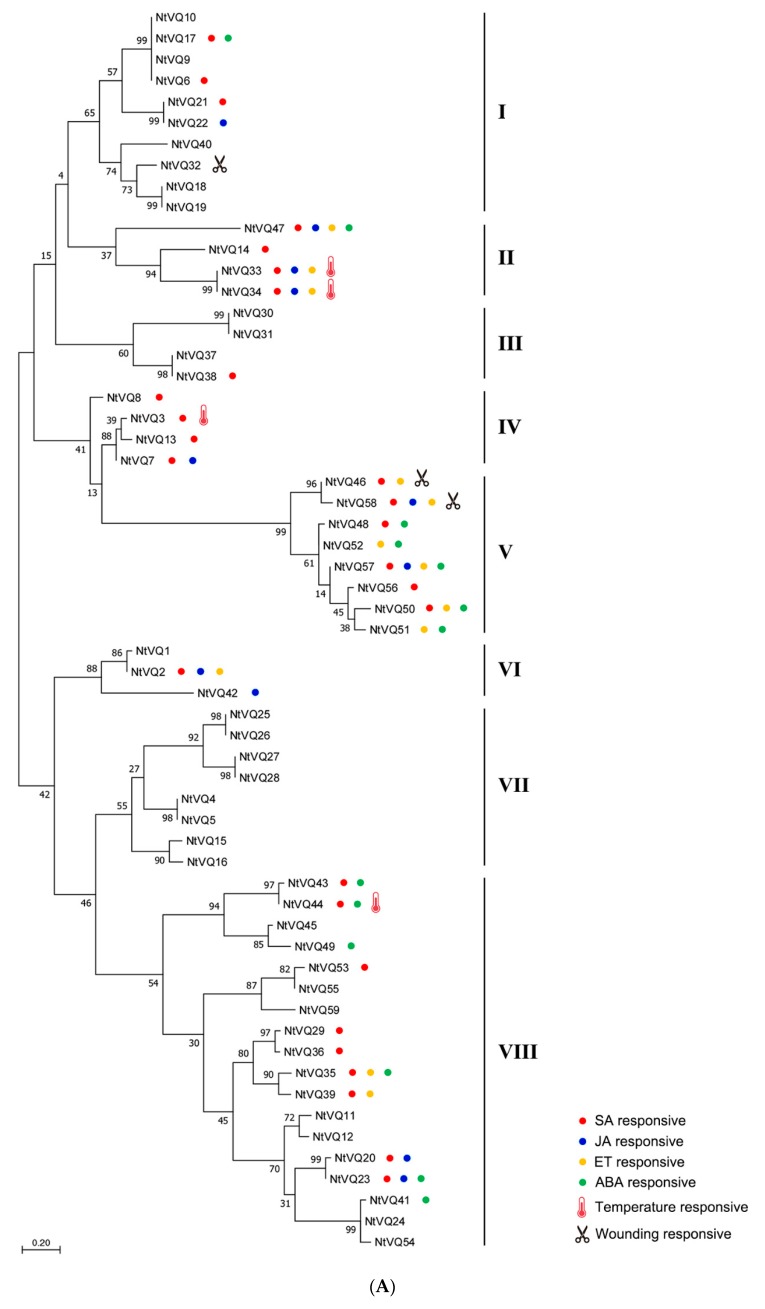

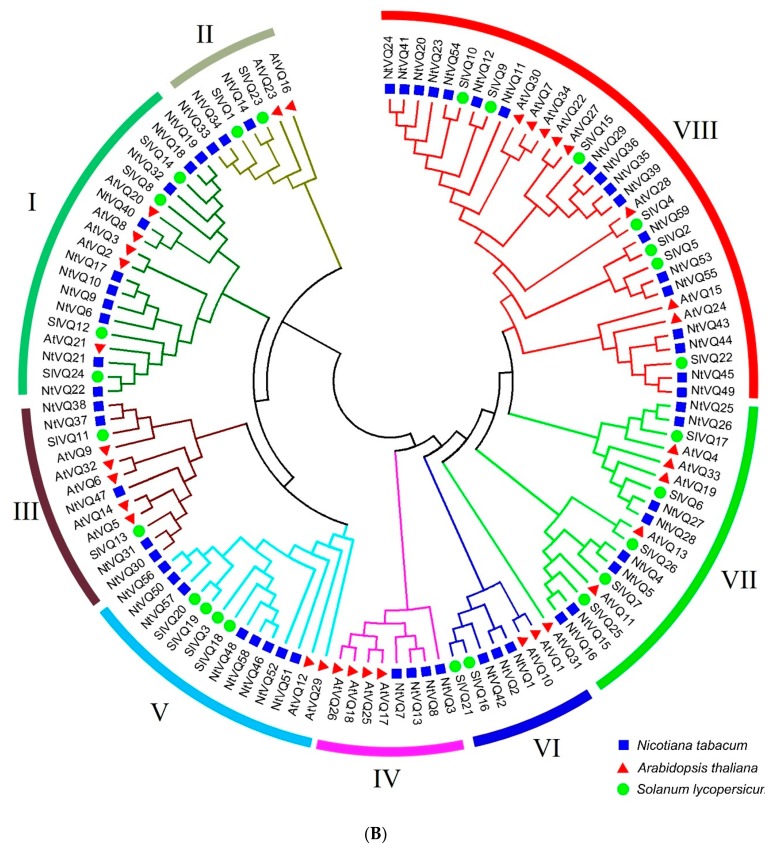
Phylogenetic tree of VQ motif-containing proteins (VQ proteins) using Maximum Likelihood method. (**A**) Phylogenetic tree and clustering of *Nicotiana tabacum* L VQ motif-containing (NtVQ) proteins in tobacco. Shown along the genes are indications of major responses to various phytohormonal and abiotic stresses. Phytohormone-responsive genes are indicated with small circles in different colors. Wounding-responsive and temperature-related genes are illustrated with a thermometer and scissors symbol, respectively. The Roman numerals I–VIII stand for separated groups in the phylogenetic tree. (**B**) Phylogenetic analysis of NtVQ proteins from tobacco, Arabidopsis and tomato. The sequences of VQ proteins in Arabidopsis and tomato were downloaded from Phytozome (https://phytozome.jgi.doe.gov/pz/portal.html, v12.1). The names of Arabidopsis and tomato VQ proteins were in line with those previously published. The phylogenetic tree of 59 tobacco NtVQs, 34 Arabidopsis AtVQs and 26 tomato SlVQs was made by Mega 7.0 using Maximum Likelihood method. Based on the clustering of the VQ proteins, tobacco NtVQs were clustered into eight subgroups (I–VIII). Proteins from tobacco, Arabidopsis and tomato are denoted by blue squares, red triangles and green circles.

Using available genomic sequences for *N. tabacum* (cultivated tobacco) in the NCBI database, we were able to data mine a total of 59 NtVQ proteins using HMMER software. Based on the homology of the NtVQ proteins to orthologs in the Pfam database, we designated these proteins as NtVQ1 to NtVQ59. To explore the evolutionary relationship of the 59 NtVQ proteins, a phylogenic tree of the 59 NtVQ proteins was constructed using the Maximum Likelihood Method in Mega 7.0 (Figure 1A). In addition, a phylogenic tree was built using the 59 NtVQ protein sequences and those of 34 and 26 VQ motif-containing proteins of Arabidopsis and tomato, respectively (Figure 1B). Phylogenetic analysis of the NtVQ proteins based upon their nucleotide sequence, gene structure (exon–intron organization) and organization of conserved protein domains showed that the tobacco NtVQ proteins cluster into eight main groups (Figure 1A). When compared with the VQ proteins across species, the *NtVQ* clustering pattern and clades in our analysis (Figure 1B) exhibited high similarities to those previously reported in Arabidopsis and tomato with only minor differences [11,24]. For example, the Arabidopsis AtVQ2 alone was clustered in one branch, while in our analysis it was clustered with AtVQ3. Moreover, the evolutionary relationship indicated that the NtVQ proteins have a close affinity with the tomato VQ proteins and have a relatively slight distant affinity with the Arabidopsis VQ proteins in the same group, since AtVQ31 was located in one branch alone. This is likely because both tomato and tobacco belong to the Solanaceae family while Arabidopsis belongs to the family of Cruciferae. Cultivated tobacco (*N. tabacum*) originated from *N. tomentosiformis* and *N. sylvestris*. Not surprisingly, results of BLAST analysis showed that the *NtVQ* genes overwhelmingly appear in pairs in the phylogenic tree with an almost evenly derived distribution from the two progenitors (i.e., 29 *NtVQ* genes from *N. tomentosiformis* and 29 from *N. sylvestris*) (Supplementary Table S2), with the only exception being NtVQ40. As discussed below, homologous genes also appear to display similar responses to both biotic and abiotic stimuli.

A variety of biochemical properties were then determined for the 59 NtVQ proteins (Supplementary Table S3). The predicted NtVQ proteins varied in length from 101 aa (NtVQ42) to 467 aa (NtVQ23), yielding proteins that ranged from 13.07 kDa (NtVQ1) to 44.68 kDa (NtVQ12) in MW. The theoretical pI of the NtVQ proteins ranged from 4.86 (NtVQ59) to 10.73 (NtVQ30 and NtVQ31), with more than half of the proteins having pIs greater than 7. To evaluate the protein hydrophobicity, a GRAVY score was defined, and the results showed that the GRAVY scores for all NtVQ proteins were negative, indicating that all of them are hydrophilic. Additionally, all but two of the NtVQ proteins (NtVQ8 and NtVQ45) had an instability index greater than 40, suggesting that most of them are potentially unstable.

Analysis of *NtVQ* gene structure showed that the vast majority (49/59 genes or 83.1%) did not contain introns (Supplementary Figure S1). Those that did contain introns (i.e., *NtVQ20*, *NtVQ23*, *NtVQ30*, *NtVQ31*, *NtVQ41*, *NtVQ46*, *NtVQ47*, *NtVQ50*, *NtVQ57,* and *NtVQ58)* each contained a single intron, which varied in size among the genes (Supplementary Figure S1). Furthermore, bioinformatics predictions of subcellular localization of the NtVQ proteins were performed, and the results showed that most NtVQ proteins were localized in the nucleus, while a few proteins were localized in the chloroplast or cytoplasm (Supplementary Table S3). The CDS of the genes and their deduced amino acids are listed in Supplementary Table S4.

**Figure 2:**
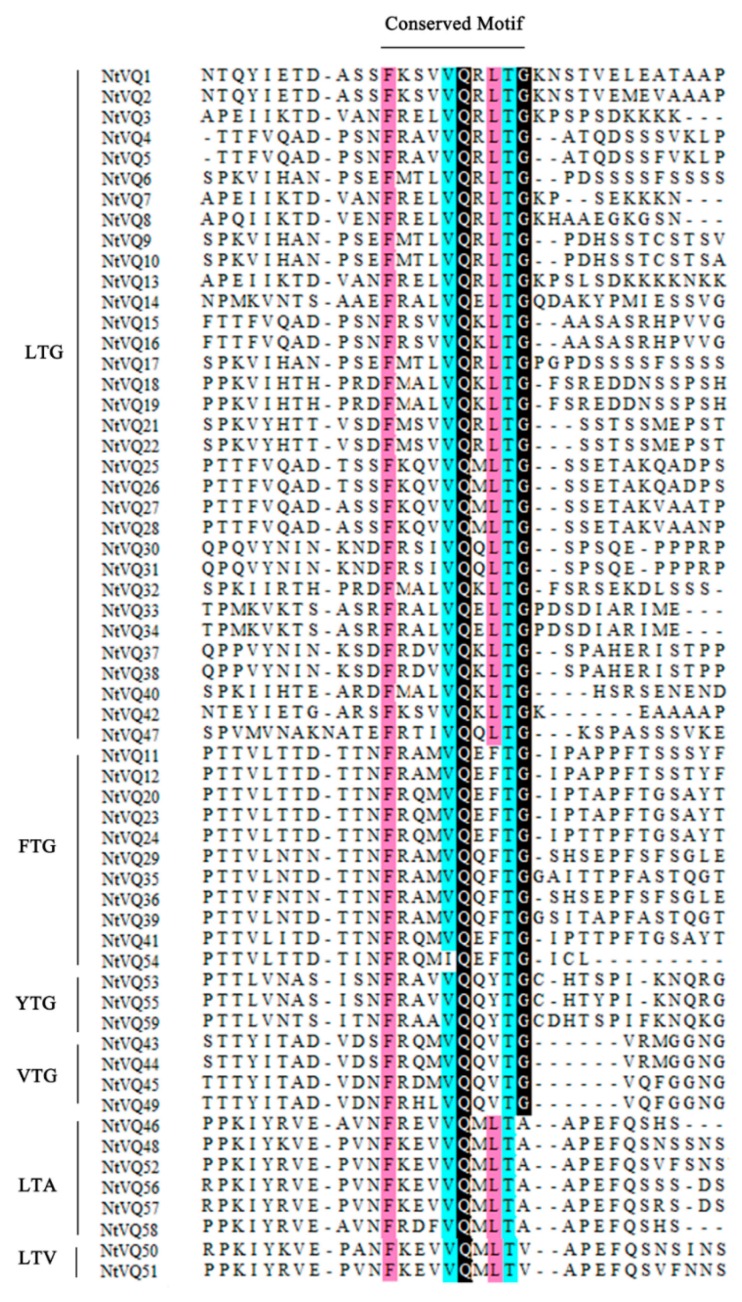
Multiple sequence alignment of the VQ motif based on their entire VQ protein length for the VQ proteins in tobacco. Multiple sequence alignment of the VQ motif for 59 NtVQ proteins showing the highly conserved FxxxVQxLTG motif, which can be further divided into several subgroups such as LTG, FTG, YTG, etc., based on the last three amino acids.

Multiple sequence alignment was performed to further investigate the differences among the NtVQ proteins. Analysis of the predicted amino acid sequence showed that all identified NtVQ proteins contained the conserved motif FxxhVQxhTG, where x represents any amino acid and h denotes a hydrophobic residue. As shown in Figure 2, six variations of the conserved VQ motif were found among the 59 NtVQ proteins: FxxxVQxLTG (33/59), FxxxVQxFTG (10/59), FxxxVQxYTG (3/59), FxxxVQxVTG (4/59), FxxxVQxLTA (6/59), FxxxVQxLTV (2/59). The most notable difference occurred in NtVQ54 where the canonical VQ core is replaced with IQ (i.e., FxxxIQxFTG). Of what functional consequence the I to V change is to the protein remains to be defined. Different clades may tend to have different conserved VQ motifs. Group V contained the LTV and LTA motif, group VIII contained YTG and FTG, while LTG sequences were shown in all the other clades.

**Figure 3:**
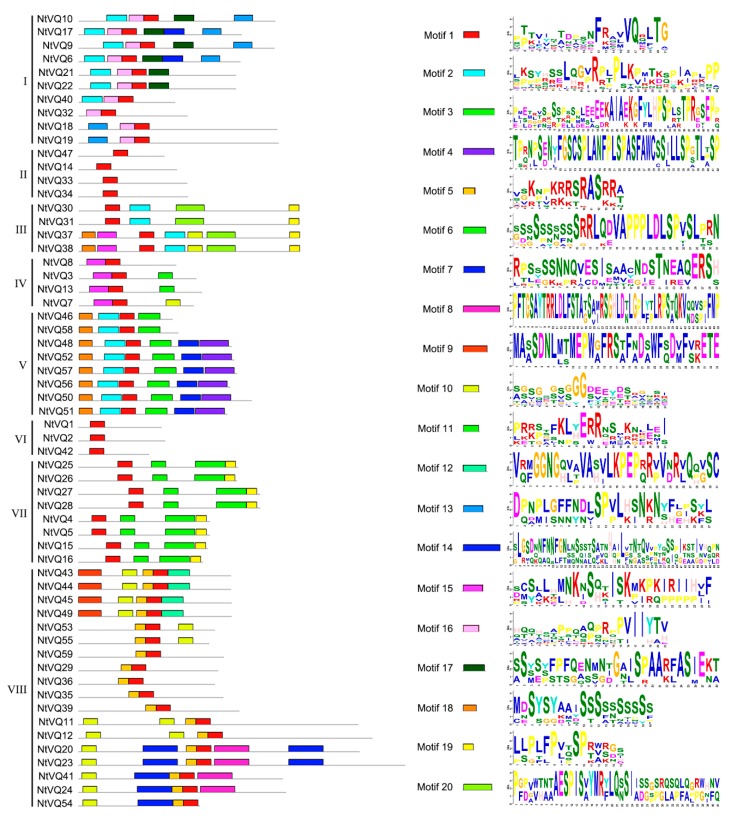
Motif composition of whole amino acid sequences in NtVQ proteins determined by MEME. Twenty motifs were identified from the complete amino acid sequences of 59 NtVQ proteins. Combinations of different motifs in each NtVQ are indicated on the left and detailed information on each motif is shown on the right.

To better understand the structural diversity of NtVQ proteins, MEME analysis was performed and a total of 20 different motifs (ranging from 15 to 50 aa in length) were identified (Figure 3). Apart from the expected conserved VQ motif found in all NtVQ proteins, each Group (I-VIII) generally contained a clearly identifiable motif structure that distinguished it from other groups. Some contained only one conserved motif and others as many as seven. For example, the Group II and VI members only contained the VQ motif, whereas two members of Group III had seven different motifs. Motif 4 was only present in Group V and motif 14 only in Group VIII. In general, VQ protein sequences belonging to the same subgroup tended to contain basically the same type of motifs (Figure 3).

### 3.2. Evolution and Divergence of the VQ Gene Family in Tobacco and Arabidopsis

To better understand the evolution and divergence of the *NtVQ* gene family, we identified 74 paralogous pairs in tobacco and 33 orthologous pairs between tobacco and Arabidopsis using Clustal Omega software and BlastP, respectively. All of the paralogous and orthologous pairs are listed in Table 1. Nine *NtVQ* genes (i.e., *NtVQ7*, *NtVQ8*, *NtVQ14*, *NtVQ32*, *NtVQ40*, *NtVQ42*, *NtVQ47*, *NtVQ54* and *NtVQ59*) failed to have any homologous genes predicted. We found that some *NtVQ* genes matched two or more *AtVQ* genes (Table 1). For example, *NtVQ6* matched *AtVQ2* and *AtVQ3*, implying the function of certain *NtVQ* genes in tobacco may be more diverse than that in Arabidopsis. We also calculated the *Ka*/*Ks* ratios of the 74 paralogous pairs in tobacco, of which 35 pairs had a *p*-value < 0.05 (see Table 2). The *Ka*/*Ks* ratio of all 35 orthologous pairs was <1, indicating that these gene pairs have undergone purifying selection pressure. In addition, the genetic differentiation of the 74 gene pairs occurred between 3 and 28 MYA, as determined using the formula T = Ks/(2 × 1 × 10^−8^) × 10^−6^.

### 3.3. Differential Expression of NtVQ Genes in Response to Phytohormonal Treatments

**Figure 4:**
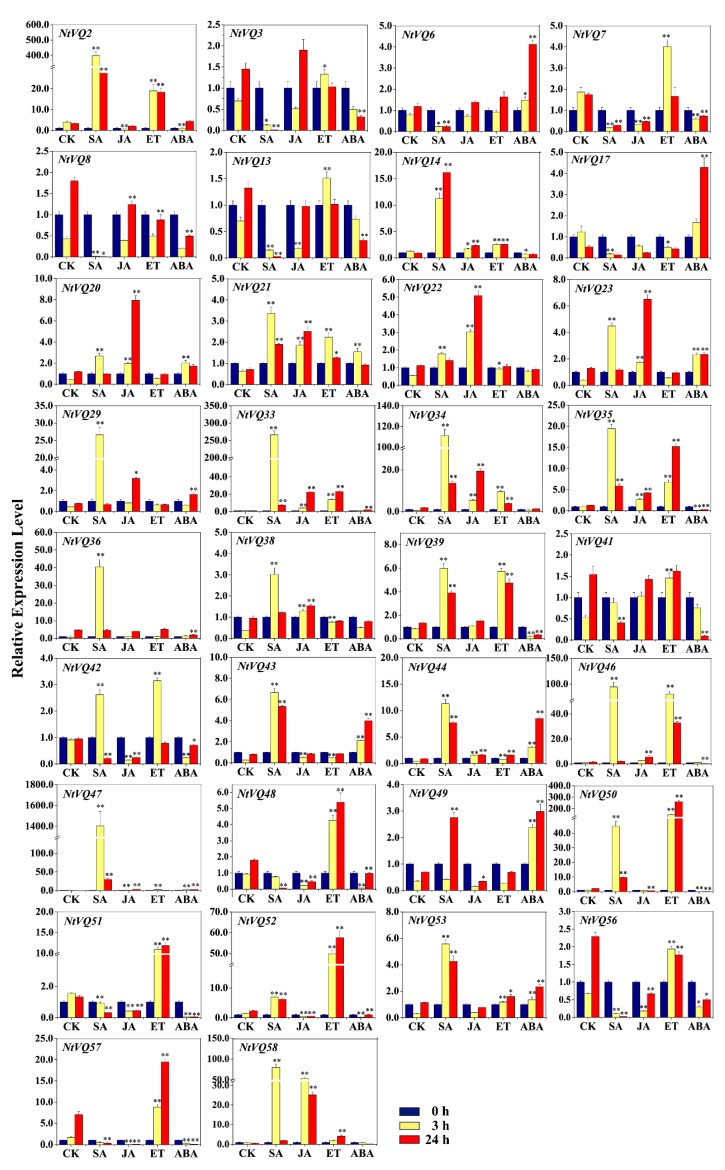
Expression analysis of significantly modulated *NtVQ* genes in response to different phytohormones. Two-week-old seedlings were treated with 2 mM SA, 100 μM JA, 1 mM ET, or 100 μM ABA for 0, 3, and 24 h before the whole seedlings were harvested for RNA isolation and qRT-PCR. The relative expression value of each *NtVQ* gene was normalized to *NtEF1α* with CK at 0 h as calibrator. CK, control group treated with 0.1% DMSO. * indicates the level of significance relative to the CK value (* *p* < 0.05, ** *p* < 0.01).

VQ proteins are known to be involved in the differential regulation of gene expression and therefore to better understand the possible roles of the various *NtVQ* gene family members, we examined their expression characteristics in tobacco seedlings before and after phytohormonal treatments. Two-week-old seedlings were treated with either SA, JA, ETH, ABA, or auxin (2,4-D) and the abundance of the various *NtVQ* gene transcripts determined by qRT-PCR at 0 h, 3 h, and 24 h after exposure. We then analyzed the relationship between phylogenetic position and phytohormonal responsiveness to determine if related genes showed similar responsiveness, which likely leads to their regulatory functions. In this analysis, we only considered genes with a transcript abundance change greater than 5-fold and a *p*-value less than 0.05. Using these criteria, we observed that while each of the seven groups contained gene family members that showed differential response to at least one phytohormone, some groups contained members that responded to a broad range of phytohormonal treatments. Among the groups whose members were broadly responsive are Groups II, IV, V, VI, and VIII (Figure 1 and Figure 4, Supplementary Figure S2, Supplementary Table S5). Interestingly, Group VII did not contain any members exhibiting differential expression (i.e., either up- or downregulation) in response to any of the phytohormonal treatments performed in this study.

Nineteen *NtVQ* genes were significantly upregulated by SA (i.e., NtVQ2, NtVQ14, NtVQ20, NtVQ21, NtVQ23, NtVQ29, NtVQ33, NtVQ34, NtVQ35, NtVQ36, NtVQ38, NtVQ39, NtVQ43, NtVQ44, NtVQ46, NtVQ47, NtVQ50, NtVQ53, and NtVQ58), with the great majority of them being significantly upregulated at 3 h and then showing a decrease in transcript abundance at 24 h (Figure 4, Supplementary Figure S2 and Table S5). The most significant change observed was the rapid and robust (~1000-fold) upregulation of NtVQ47 at 3 h post-treatment. Nine NtVQs (i.e., NtVQ3, NtVQ6, NtVQ7, NtVQ8, NtVQ13, NtVQ17, NtVQ48, NtVQ56, and NtVQ57) were substantially downregulated in response to SA and they were located in Groups I, IV, and V.

In our analysis of JA treatment, seven genes (*NtVQ20*, *NtVQ22*, *NtVQ23*, *NtVQ33*, *NtVQ34*, *NtVQ47,* and *NtVQ58*) were significantly upregulated at both 3 h and 24 h, while four genes (*NtVQ2*, *NtVQ7*, *NtVQ42,* and *NtVQ57*) were significantly downregulated at 3 h and 24 h. Notably, the transcript level of *NtVQ58* was induced more than 40 times at both 3 h and 24 h (Figure 4, Supplementary Figure S2 and Table S5).

*NtVQ* genes in Groups II and V were among the most significantly upregulated family members in response to ETH treatment, including *NtVQ2*, *NtVQ33*, *NtVQ34*, *NtVQ35*, *NtVQ39*, *NtVQ42*, *NtVQ46*, *NtVQ47*, *NtVQ50*, *NtVQ51*, *NtVQ52*, *NtVQ57,* and *NtVQ58*. Groups V and VIIII contained the greatest number of genes responsive to ABA treatment (Figure 4, Supplementary Figure S2 and Table S5). Under ABA treatment, six genes (*NtVQ17*, *NtVQ23*, *NtVQ43*, *NtVQ44, NtVQ47,* and *NtVQ49*) were induced at both 3 h and 24 h, while six genes (*NtVQ35*, *NtVQ48*, *NtVQ50*, *NtVQ51*, *NtVQ52,* and *NtVQ57*) were repressed at both 3 h and 24 h, with *NtVQ50* and *NtVQ51* exhibiting more than 10-fold downregulation relative to control. Two genes (*NtVQ41* and *NtVQ46*) were slightly induced at 3 h and repressed significantly at 24 h.

One interesting observation is that very closely related genes (e.g., *NtVQ33*/*NtVQ34* in II group, *NtVQ46*/*NtVQ58* and *NtVQ48*/*NtVQ52* in Group V, and *NtVQ35/NtVQ39* and *NtVQ20/NtVQ23* in Group VIII) all appeared to be similarly regulated (also summarized in Figure 1). This likely reflects the fact that they are paired genes from different progenitor backgrounds that are involved in similar developmental or stress responsive regulatory pathways.

### 3.4. NtVQ Gene Expression Following Abiotic Treatments

**Figure 5:**
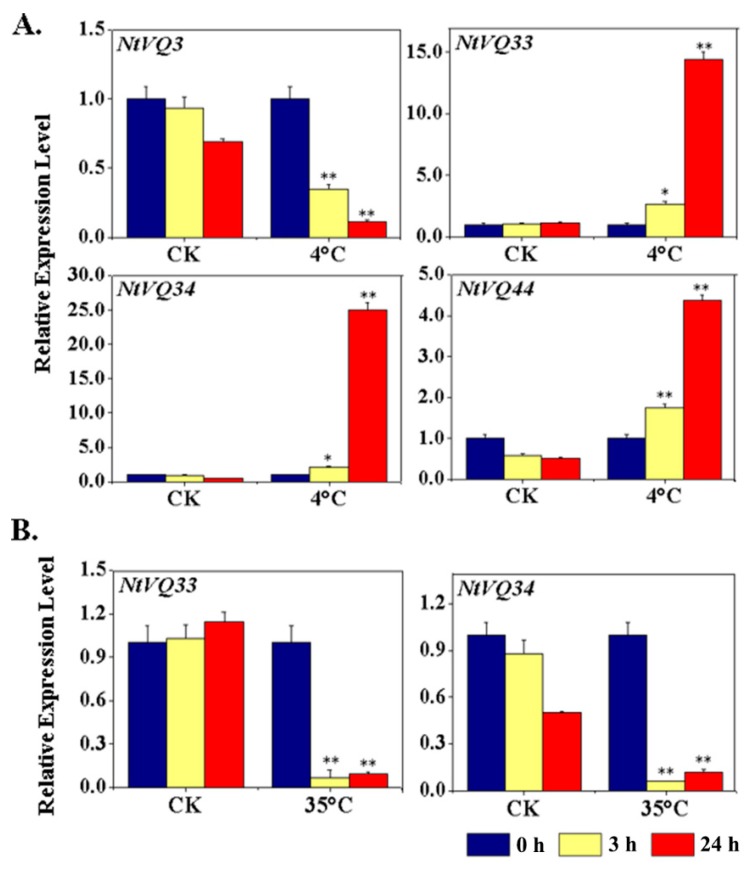
Expression analysis of significantly modulated *NtVQ* genes in response to cold and heat treatment. Expression analysis of *NtVQ* genes in response to low temperature at 4 °C (**A**), and to high temperature at 35 °C (**B**). Two-week-old seedlings were treated with cold or heat and collected at indicated time points, respectively. The relative expression value of each *NtVQ* gene was normalized to *NtEF1α* with CK at 0 h as calibrator. CK, control group grown constantly at 25 °C. * indicates the level of significance relative to the CK value (* *p* < 0.05, ** *p* < 0.01).

**Figure 6:**
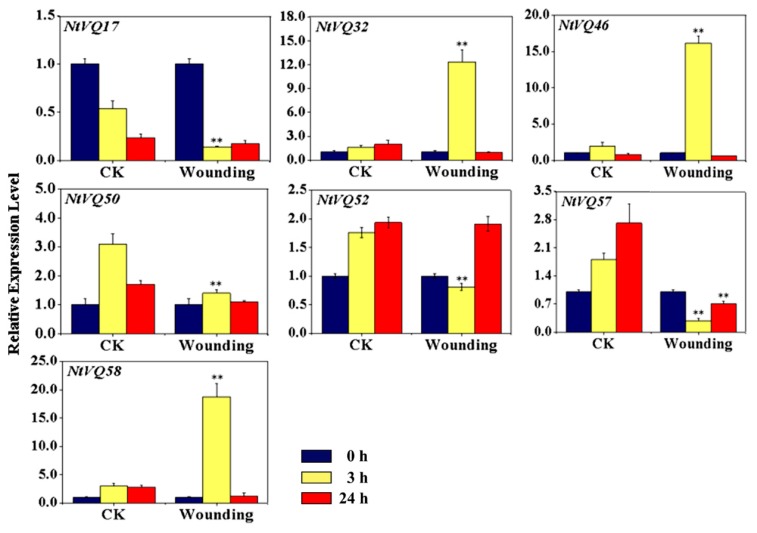
Expression analysis of significantly modulated *NtVQ* genes in response to mechanical wounding. Two-week-old seedlings were treated with wounding for 0, 3 and 24 h, respectively, before collected for RNA isolation and qRT-PCR. The relative expression value of each *NtVQ* gene was normalized to *NtEF1α* with CK at 0 h as calibrator. CK, control group without wounding. * indicates the level of significance relative to the CK value (* *p* < 0.05, ** *p* < 0.01).

We also examined the expression of the various members of the *NtVQ* family in response to different abiotic stresses, such as heat (35 °C) and cold (4 °C) treatment and response to mechanical wounding. As shown in Figure 5 (also Supplementary Figures S3 and S4) the expression of four *NtVQ* genes was significantly altered in response to cold treatment, with *NtVQ33*, *NtVQ34,* and *NtVQ44* being significantly induced and *NtVQ3* being repressed. Intriguingly, the expression levels of *NtVQ33* and *NtVQ34* were also found to be significantly suppressed at 3 h and 24 h post heat treatment, suggesting a close relationship of these two genes with temperature sensing and responses. Additionally, three genes (*NtVQ32*, *NtVQ46,* and *NtVQ58*) were significantly transiently induced by mechanical wounding showing highly elevated levels of transcript 3 h post-treatment and then a return to baseline by 24 h (Figure 6).

### 3.5. NtVQ Gene Expression Following Pathogen Infection

**Figure 7:**
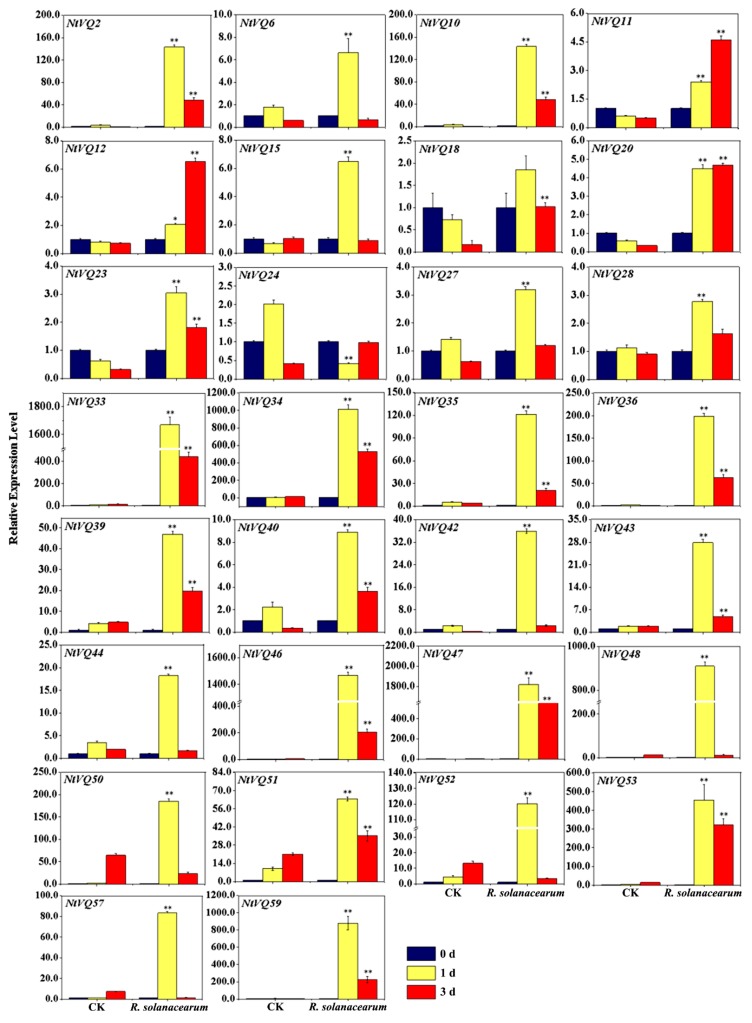
Expression analysis of significantly modulated *NtVQ* genes in response to challenge by *Ralstonia solanacearum*. Four-week-old seedlings were inoculated with 50 μL bacteria (OD = 0.001) for 0, 1 and 3 d before the leaf tissues were harvested for RNA extraction. The relative expression value of each *NtVQ* gene was normalized to *NtEF1α* with CK at 0 h as calibrator. CK, control group inoculated with 10 mM MgCl_2_. * indicates the level of significance relative to the CK value (* *p* < 0.05, ** *p* < 0.01).

To assess the roles of *NtVQ* genes in controlling the resistance response of tobacco to bacterial pathogens, we inoculated young tobacco leaves with a tobacco wilt-causing bacterium *R. solanacearum* (*Rsc*) and determined which genes showed the greatest expression response. Somewhat surprisingly, ~51% (30/59) of the gene family members showed a significant level of induction following the bacterial pathogen challenge, exhibiting elevated levels of transcripts 1-day post-inoculation (Figure 7). In most cases the induced transcript levels started to drop or even return to near baseline levels by 3 days post-inoculation. Particularly, *NtVQ11*, *NtVQ12,* and *NtVQ20* had transcript levels that were higher at day 3 post-inoculation than at day 1 (Figure 7).

### 3.6. Analysis of Putative cis-Regulatory Elements in the Promoters of the NtVQ Gene Family Members

Having determined the general expression characteristics of various *NtVQ* genes in response to phytohormonal treatment, abiotic and biotic stresses, we sought to determine whether it was possible to identify *cis*-regulatory elements in the promoters of these genes that could potentially be responsible for directing their differential activation or repression. Therefore, we analyzed the ~2 kb nucleotide sequence 5′ upstream of the predicted start codon (i.e., ATG) for each *NtVQ* gene using the PlantCARE software. In addition to the well characterized TATA and CAAT boxes, three categories of *cis*-regulatory elements were found to be highly represented in the promoter region of *NtVQ* genes (Supplementary Table S6). The first category comprises “light responsive elements,” such as the Box4, G-box, GATA-motif, I-box, etc. Almost all *NtVQ* genes contained at least one light-responsive element in their promoter regions (Supplementary Table S6).

The second category is composed of phytohormone-responsive elements, such as the CGTCA-motif, TGACG, TCA-element, ABRE, and ERE. The presence of these elements correlated loosely with the described phytohormonal responsiveness of the *NtVQ* gene, but there were also strong correlations. For example, the promoter region of *NtVQ43* contained ABRE, TCA-element, TC-rich repeats, which are *cis*-regulatory elements associated with ABA responsiveness, SA responsiveness, and stress and defense responsiveness, respectively (Supplementary Table S6). In our studies described above, *NtVQ43* was indeed found to be significantly induced by SA, ABA, and *Rsc* treatments (Figure 4 and Figure 7). Other examples of a strong correlation between the presence of *cis*-regulatory elements for phytohormonal responsiveness and stress response and documented gene expression activation by these stimuli were also seen in *NtVQ7*, *NtVQ23*, *NtVQ29*, *NtVQ35*, *NtVQ42*, *NtVQ44,* and *NtVQ46*. Nearly half of promotors of the genes that significantly responded to *Rsc* contained at least one TC-rich repeat, which is a *cis*-acting element involved in defense and stress responsiveness (Supplementary Table S6).

The third category consisted of *cis*-regulatory elements associated with response to external or environmental stresses. This category includes TC-rich elements, wound response elements (e.g., WUN-motif), and low temperature response (LTR) elements. As might be predicted based on their strong induction in response to cold treatment, the promoter region of *NtVQ33* had at least one LTR element, and *NtVQ32* and *NtVQ58* contained one or more WUN-motifs consistent with their induction by mechanical wounding (Figure 5 and Figure 6, Supplementary Table S6). In a few cases, such as *NtVQ57*, the promoter region contained multiple copies of the LTR element, but the gene showed very low level of response to cold temperature. In addition, 34 out of 58 *NtVQ* gene promoters contained one or more W-box motif, which is binding site for WRKY transcription factor.

## 4. Discussion

The VQ motif-containing proteins have been shown to be major transcriptional regulators in plants, working independently or in combination with other TFs to regulate diverse growth and developmental processes and responses to biotic and abiotic stresses [5,10]. However, there is limited information on the characterization of VQ motif-containing protein in *N*. *tabacum*. Therefore, the comprehensive analysis of *NtVQ* genes and their expression pattern under various abiotic, biotic, and phytohormonal treatments could be beneficial to further understanding the mechanisms of plant growth and development, as well as aiding in the selection of candidate genes for deeper functional characterization.

### 4.1. In-Silico Analysis of NtVQ Genes

In this work, we found that the VQ protein gene family in tobacco is comprised of 59 members comparable in size and organization to that reported in other plants, such as Arabidopsis (34 members) [11], rice (39 members) [12], and apple (49 members) [20]. The number of *NtVQ* genes predicted was lower than expected, given the fact that tobacco is an allotetraploid with a 3.6 Gb genome size, whereas Arabidopsis, rice, and apple are diploids with genome sizes of 125 Mb [50], 389 Mb [51] and 651 Mb [52], respectively. Thus, it appears that there is no direct relationship between the numbers of *VQ* genes and the genome size. Jiang et al. [26] found that segmental duplication was regarded as the main mechanism for *VQ* gene expansion, so the evolutionary process may explain the specific *VQ* gene numbers for one species rather than the size of genome. In addition, we could not rule out the possibility that additional *VQs* are present in the genome but were not detected as a result of gaps in the genome assembly or incomplete/wrong gene prediction. Cultivated tobacco (*N. tabacum*) (2n = 4χ = 48) evolved from the interspecific hybridization of the ancestors of *N. sylvestris* (2n = 24, maternal donor) and *N. tomentosiformis* (2n = 24, paternal donor) about 200,000 years ago [53,54]. The *Ks* value of each paralogous pair was used to find gene duplication events, and this analysis revealed that most duplication events of the *NtVQ* genes occurred between 3 and 28 MYA. This date precedes the estimated date for the origin of cultivated tobacco formation, suggesting these genes duplication events likely occurred in the ancestral forms and were carried forward. The *Ka/Ks* ratios in the various gene pairs are different, and *Ka/Ks* ratios of all the gene pairs are less than 1, suggesting these gene pairs underwent purifying selection pressure. The above analysis indicates that VQ proteins were highly conserved during evolution and evolved slowly, similar to what was reported on the evolutionary rates of VQ proteins in soybean [22].

Structural predictions indicated that most *NtVQ* genes (83.1%) in tobacco lack introns (Supplementary Figure S1), consistent with VQ protein gene structure in other plants (e.g., 88.2%, 90%, and 92.3% of the VQ protein genes in Arabidopsis [11], Chinese Cabbage [14], and tomato [24], respectively, lack introns). It was reported that a large number of introns might be lost in *VQ* genes during evolution [18]. The distribution of *NtVQ* genes among clades based on protein sequence conservation appears to follow the pattern observed in other plant species as well. Analysis of the phylogenic tree of tobacco VQ proteins has shown that the clades are similar to those described for Arabidopsis [11] and tomato [24]. Tobacco NtVQ protein has an average of 223.5 amino acids, which is similar to the known VQ protein in many plants, where most of the *VQ* genes encode fewer than 300 amino acids, including Arabidopsis [11], rice [12], maize [16], Moso bamboo [18], apple [20], poplar [15], Chinese Cabbage [14], soybean [22], tomato [24], tea [23], and so on. On the contrary, four genes (*NtVQ11*, *NtVQ12*, *NtVQ20,* and *NtVQ23*) encode protein of more than or equal to 400 amino acids. Furthermore, in silico prediction of the subcellular localization of the encoded NtVQ proteins showed that the majority localize to the nucleus (Supplementary Table S3), which is similar to many known plants, such as Arabidopsis [11] and tea [23]. Thus, this VQ family appears to be a relatively conserved family evolutionarily. There are six variations of the conserved VQ motif among the 59 NtVQ proteins: FxxxVQxLTG (33/59), FxxxVQxFTG (10/59), FxxxVQxVTG (4/59), FxxxVQxYTG (3/59), FxxxVQxLTA (6/59), FxxxVQxLTV (2/59). In previous studies, it was known that there are six types of motifs in Arabidopsis (LTG, FTG, VTG, YTG, LTS, and LTD) [11], six types in maize (LTG, FTG, VTG, ITG, ATG, and LTA) [16], four types in rice (LTG, FTG, VTG, and ITG) [13], six types in Chinese cabbage (LTG, FTG, VTG, YTG, LTV, and LTS) [14], five types in Moso bamboo (LTG, FTG, VTG, ITG, and LTA) [18], five types in soybean (LTG, FTG, VTG, LTS, and LTR) [22], four types in apple(LTG, FTG, VTG, and LTC) [20], three types in grapevine (LTG, FTG, and VTG) [13] and seven in tomato (LTG, FTG, VTG, LTS, LTA, YTG, and HTG) [24]. Comparing the different variations of conserved VQ domain in the above plant species, we can see LTG, FTG, and VTG are the three most common ones for the VQ domain in plants. Tobacco does not contain a unique variation of conserved VQ domain and the variations of conserved VQ domain in tobacco have also been seen in the above plant species. Even though tomato and tobacco belong to the same family, they do not tend to have the same conserved VQ domain variation. Therefore, the variations of conserved VQ motif may be restricted to specific species and the types and numbers of the variations of VQ motif vary in different species. Intriguingly, the LTV and LTA motif-containing sequences all belong to clade V, the VTG, YTG, and FTG sequences to clade VIII, and the LTG sequences are spread in all the other clades, demonstrating the genes clustering in the same clade of the phylogenetic tree may have similar gene structure.

The predicted NtVQ proteins all contain the conserved VQ domain, with the exception of NtVQ54, in which an isoleucine replaces the canonical valine in the conserved domain (Figure 2). VQ proteins with altered core domains have also been found in the rice and maize gene families, where the VQ core amino acids of OsVQ37 and OsVQ39 in rice and ZmVQ15, ZmVQ28, and ZmVQ58 in maize are replaced by a VH core amino acids [12,16]. It was reported the VH core amino acids only showed in monocot plants, this may claim the difference between monocot and dicots during the evolutionary process in history. Hence, it is reasonable for us to deduce that the presence of IQ core amino acids may highlight some difference or unique features between tobacco and other species. This may be explained by functional diversity between *VQ* gene family members in different species during the evolution in history, since the VQ motif-containing family has an ancient origin [26].

MEME analysis showed that the VQ protein sequences belonging to the same subgroup contained basically the same type of motifs (Figure 3), indicating that close proteins may share similar structure and function. Notably, motif 1 corresponds to the VQ-containing motif, the common one in all NtVQ proteins, and may give specific biological function to VQ proteins in tobacco plants. These results are similar to the findings in tomato [24] and soybean [22]. Furthermore, the similarities in the motif composition of the NtVQ proteins are consistent with the results of the phylogenetic analysis, and the distinctions among the different groups/subgroups indicate that the function of the NtVQ members varies [16,22]. In addition, instability index analysis showed most of the NtVQ proteins are unstable (Supplementary Table S3), indicating their expression is likely influenced by external stimuli, which is consistent with their roles as small regulatory proteins. The activation of *NtVQ* genes were proved by the following abiotic and biotic treatments.

### 4.2. Expression Analysis of NtVQ Genes in Response to Biotic and Abiotic Stresses

Previous studies have shown that members of the *VQ* gene family are differentially expressed throughout plant development and in response to various endogenous and exogenous cues. Molecular genetic evidence has shown several plant VQ proteins likely to be important regulators against plant disease resistance and stress tolerance. In this study, we did a general analysis of *NtVQ* genes in defense responses and disease resistance responses of tobacco plants using qRT-PCR analysis. Expression of a substantial percent of *NtVQ* genes (~57.6%, 34 out of 59 genes) was responsive to a number of phytohormones associated with plant defense and stress response (Figure 1 and Figure 4, Supplementary Figure S2, Supplementary Table S5). We found that the *NtVQ* genes were more responsive to SA treatment, where the numbers of induced genes were more than those under other phytohormonal treatments, suggesting these genes might play a role in SA signaling pathway (Figure 1 and Figure 4, Supplementary Figure S2, Supplementary Table S5). This result is consistent with previous studies that most *GmVQ* genes can repond to SA treatment in soybean [17,22]. Similar cases for *VQ* genes under SA treatment were also reported in other species, such as Arabidopsis [11], grapevine [13], and Chinese cabbage [14]. In Arabidopsis, the expression levels of many SA-induced *VQ* genes peaked at early hours (e.g., 4 h post treatment) and some of them started to decline as time progressed further (e.g., 12 h or particularly 24 h post treatment) [11]. Similarly, most of the SA-upregulated *NtVQ* genes, including *NtVQ2*, *NtVQ20, NtVQ21, NtVQ23, NtVQ29, NtVQ33*, *NtVQ34*, *NtVQ35*, *NtVQ36, NtVQ38, NtVQ39, NtVQ43*, *NtVQ44*, *NtVQ46, NtVQ47*, *NtVQ50, NtVQ53,* and *NtVQ58*, exhibited tendencies of decrease at 24 h compared to 3 h. Nevertheless, our finding is not consistent with that of *VQ* genes in Chinese cabbage, where 17 of 44 *BrVQ* genes were upregulated by SA and most of the induced genes (10/17) kept the going-up trend even at 24 h post SA treatment [14]. Therefore, *VQ* genes in different species may respond to SA treatment differently and their expression pattern may vary.

For *Rsc* treatment, 30 out of 59 (~50.85%) *NtVQs* were upregulated after *Rsc* infection, suggesting putative implications of these genes in the tobacco defense response (Figure 7). This result is consistent with previous studies that most *VQ* genes can respond to pathogens in Arabidopsis [11], rice [55], and strawberry [56]. Plant hormones have been directly related to or implicated in plant defense responses to a variety of microbial pathogens, and it has been reported that SA-mediated host immunity plays a crucial role in combating bacteria in plants [57]. Our results showed many *NtVQ* genes responsive to SA 24 h post treatment were also induced upon *Rsc* treatment, including *NtVQ*2, *NtVQ33*, *NtVQ34*, *NtVQ35*, *NtVQ39*, *NtVQ43*, *NtVQ44*, *NtVQ47*, *NtVQ50,* and *NtVQ53* (Figure 7), implying that these genes may be involved in pathogen defense. Studies have also shown that SA and JA signaling pathway are mutually antagonistic, and genes involved in plant defense often play opposite roles in resistance to two distinct types of plant pathogen, i.e., SA-mediated defense often mediates plant defense against biotrophic pathogens, whereas JA is important for resistance to necrotrophic pathogens [11]. Analysis of the differently expressed *NtVQ* genes responsive to SA, JA, and *Rsc* treatments revealed four genes (i.e., *NtVQ2*, *NtVQ39*, *NtVQ43,* and *NtVQ53*) were highly induced by SA and *Rsc* but not induced or slightly repressed by JA. It is speculated that these four genes are likely to play roles in tobacco defense against *Rsc* and are worth further investigation.

ETH was thought to be an important factor inducing basic pathogen-related (PR) genes [58]. The crosstalk between JA and ETH is complicated and intriguing. It was reported that the JAZ inhibition on EIN3/EIL1 mediates JA and ETH signaling synergy in plant resistance to necrotrophic fungi, whereas the reciprocal counteraction between MYC2 and EIN3/EIL1 mediates the JA and ETH signaling antagonism in hook formation, wound-responsive gene expression, and defense against generalist herbivores [59]. Our results showed that *NtVQ47* responded to both JA and ETH the same way at both 3 h and 24 h (Figure 1 and Figure 4, Supplementary Figure S2, Supplementary Table S5), and thus it may play a role in plant resistance to necrotrophic pathogens. In addition, *NtVQ57* responded to both JA and ET differently at both 3 h and 24 h, and this gene slightly responded to mechanical wounding treatment, as well. Hence, *NtVQ57* may have a similar function to EIN3/EIL1. The highly induced or decreased genes like *NtVQ58* by JA treatment and the *NtVQ50/NtVQ51* pair by ABA treatment are likely to play an important role in JA or ABA pathway.

Abscisic acid (ABA) is an important phytohormone and plays a critical role in the response to various abiotic stress signals. In this study, we found that six *NtVQs* (*NtVQ17*, *NtVQ23*, *NtVQ43*, *NtVQ44, NtVQ47,* and *NtVQ49*) significantly upregulated at both 3 h and 24 h, and six *NtVQs* (*NtVQ35*, *NtVQ48*, *NtVQ50*, *NtVQ51*, *NtVQ52,* and *NtVQ57*) significantly downregulated at both 3 h and 24 h (Figure 1 and Figure 4, Supplementary Figure S2, Table S5). Previously, it was reported that only three genes showed upregulation and multiple *OsVQ* genes showed downregulation after 12 h under ABA treatment in rice [12], whereas most of the *VQ* genes were upregulated when exposed to ABA treatment in Moso bamboo [18] and *Eucalyptus grandis* [60]. These different expression patterns of the *VQ* gene family upon ABA treatment in different plant species may indicate diverse functions of *VQ* genes towards stress.

Furthermore, we assessed the expression levels of the *NtVQ* genes in tobacco plants in response to temperature change and wounding. These studies showed that several *NtVQ* genes (*NtVQ3*, *NtVQ33*, *NtVQ34,* and *NtVQ44*) were significantly regulated by cold or heat treatment. The limited response of members of the NtVQ family contrasts with the observation that 56% (14/25) of the *VQ* genes in soybean were upregulated in response to cold treatment [22]. It is worth noting that the *N. tomentosiformis*-derived *NtVQ33* and *N. sylvestris*-derived *NtVQ34* genes were both differentially temperature responsive, with their expression increasing in response to cold treatment and decreasing in response to heat treatment (Figure 5). Interestingly, both *NtVQs* were also significantly upregulated to similar extents by SA, JA, and ET (Figure 4), suggesting that these genes may play a pivotal role in integrating components of the phytohormone and temperature stress response signal transduction pathways.

Among the 59 *NtVQ* genes, three (*NtVQ32*, *NtVQ46,* and *NtVQ58*) were rapidly induced by mechanical wounding and their expression returned to a basal level by 24 h post-treatment (Figure 6). Wound responsiveness is generally associated with signal transduction mediated by one or more phytohormones (e.g., JA, ETH, and ABA). It is very interesting that the responsiveness of *NtVQ32* to wounding is independent of any of the phytohormones tested. However, *NtVQ46* was highly induced by SA and ETH, *NtVQ58* was highly induced by SA and JA. This is the first evidence showing that the *VQ* gene family can be induced by wounding treatments in plants.

For the promoter analysis, multiple *cis*-regulatory elements that relate to phytohormone and abiotic or biotic stressors were identified in the promoter region of *NtVQ* genes. Similarly, most of these *cis*-elements occurred in other plants, like grapevine [13], maize [16], and soybean [22]. Although some promoters of *NtVQ* genes contain auxin-related elements (Supplementary Table S6), the expression of these genes was not significantly induced by 2,4-D (Supplementary Table S5). It was proposed in Arabidopsis that VQ proteins were likely to play a crucial role in plant growth, development, and response to environmental stresses, acting as cofactors of Group I and IIc WRKY TFs [11]. In our analysis, 34 out of 58 *NtVQs* (60.34%) contained one or more W-box motif (TTGAC(C/T), the WRKY-binding sites) in their 1.5 Kb promoter regions. This percentage varies in different species, as 78% *VvVQ* genes in grapevine [13], 91% *ZmVQ* genes in maize [16], and 30.67% *GmVQ* genes in soybean [22] contain the W-box motif in their promoter regions, respectively. The average frequency of the W-boxes in the 1.5 Kb promoter regions of the 34 Arabidopsis *VQ* genes was approximately 3.8 [61], while that of the tobacco *NtVQ* genes was approximately 0.68 (Supplementary Table S6). This is likely to suggest a lower level of transcriptional interaction between *WRKY* and *VQ* genes in tobacco compared to Arabidopsis.

Among the most studied regulatory activities of VQ proteins is their interaction with the WRKY class of TFs [13,62,63]. WRKYs contain almost invariant WRKYGQK motif at the N-terminus followed by a Cx_4-5_Cx_22-23_HxH or Cx_7_Cx_23_HxC zinc-finger motif [64,65]. Recent evidence indicates that VQ proteins interact with WRKY TFs via the conserved V and Q residues, and that the amino acid residues flanking the core VQ motif are also required for the interaction with the WRKY domains and are important regions for the specificity of VQ–WRKY binding [20,37]. Many studies have shown the interactions among various VQ proteins and WRKY TFs to be significantly important for the growth and development of plants, such as the interactions between AtVQ9 and WRKY8 [28], AtVQ14 and MINISEED3/WRKY10 [29], AtVQ20 and WRKY2 and 34 [31], AtVQ21 and WRKY25 and 33 [32,33], AtVQ22 and WRKY28 and 51 [35,66], as well as AtVQ23 and WRKY33 [37]. Although evidence of the interactions between VQ proteins and WRKYs in tobacco is limited, it is of considerable significance to functionally characterize these small proteins in tobacco due to the important roles they are potentially playing during plant growth and development.

The results of this analysis provide the first comprehensive characterization of *NtVQ* genes in tobacco and a foundation for further detailed studies aiming at functional characterization of the different regulatory networks underlying their role in plant developmental and stress-related processes.

## Figures and Tables

**Table 1:** Paralogous (Nt-Nt) and orthologous (Nt-At) gene pairs.

Nt-Nt	Nt-Nt	Nt-At
*NtVQ1/NtVQ2*	*NtVQ48/NtVQ51*	*NtVQ2/AtVQ1*
*NtVQ2/NtVQ1*	*NtVQ48/NtVQ52*	*NtVQ6/AtVQ2*
*NtVQ3/NtVQ13*	*NtVQ48/NtVQ56*	*NtVQ6/AtVQ3*
*NtVQ4/NtVQ5*	*NtVQ48/NtVQ57*	*NtVQ27/AtVQ4*
*NtVQ5/NtVQ4*	*NtVQ49/NtVQ45*	*NtVQ31/AtVQ5*
*NtVQ6/NtVQ17*	*NtVQ50/NtVQ48*	*NtVQ30/AtVQ6*
*NtVQ9/NtVQ10*	*NtVQ50/NtVQ51*	*NtVQ41/AtVQ7*
*NtVQ10/NtVQ9*	*NtVQ50/NtVQ52*	*NtVQ40/AtVQ8*
*NtVQ11/NtVQ12*	*NtVQ50/NtVQ56*	*NtVQ38/AtVQ9*
*NtVQ12/NtVQ11*	*NtVQ50/NtVQ57*	*NtVQ2/AtVQ10*
*NtVQ13/NtVQ3*	*NtVQ51/NtVQ48*	*NtVQ4/AtVQ11*
*NtVQ15/NtVQ16*	*NtVQ51/NtVQ50*	*NtVQ56/AtVQ12*
*NtVQ16/NtVQ15*	*NtVQ51/NtVQ52*	*NtVQ27/AtVQ13*
*NtVQ17/NtVQ6*	*NtVQ51/NtVQ56*	*NtVQ30/AtVQ14*
*NtVQ18/NtVQ19*	*NtVQ51/NtVQ57*	*NtVQ45/AtVQ15*
*NtVQ19/NtVQ18*	*NtVQ52/NtVQ48*	*NtVQ14/AtVQ16*
*NtVQ20/NtVQ23*	*NtVQ52/NtVQ50*	*NtVQ7/AtVQ17*
*NtVQ21/NtVQ22*	*NtVQ52/NtVQ51*	*NtVQ7/AtVQ18*
*NtVQ22/NtVQ21*	*NtVQ52/NtVQ56*	*NtVQ26/AtVQ19*
*NtVQ23/NtVQ20*	*NtVQ52/NtVQ57*	*NtVQ18/AtVQ20*
*NtVQ24/NtVQ41*	*NtVQ53/NtVQ55*	*NtVQ6/AtVQ21*
*NtVQ25/NtVQ26*	*NtVQ55/NtVQ53*	*NtVQ53/AtVQ22*
*NtVQ26/NtVQ25*	*NtVQ56/NtVQ48*	*NtVQ14/AtVQ23*
*NtVQ27/NtVQ28*	*NtVQ56/NtVQ50*	*NtVQ49/AtVQ24*
*NtVQ28/NtVQ27*	*NtVQ56/NtVQ51*	*NtVQ13/AtVQ25*
*NtVQ29/NtVQ36*	*NtVQ56/NtVQ52*	*NtVQ7/AtVQ26*
*NtVQ30/NtVQ31*	*NtVQ56/NtVQ57*	*NtVQ53/AtVQ27*
*NtVQ31/NtVQ30*	*NtVQ57/NtVQ48*	*NtVQ53/AtVQ28*
*NtVQ33/NtVQ34*	*NtVQ57/NtVQ50*	*NtVQ57/AtVQ29*
*NtVQ34/NtVQ33*	*NtVQ57/NtVQ51*	*NtVQ26/AtVQ31*
*NtVQ35/NtVQ39*	*NtVQ57/NtVQ52*	*NtVQ30/AtVQ32*
*NtVQ36/NtVQ29*	*NtVQ57/NtVQ56*	*NtVQ25/AtVQ33*
*NtVQ37/NtVQ38*	*NtVQ58/NtVQ46*	*NtVQ11/AtVQ34*
*NtVQ38/NtVQ37*		
*NtVQ39/NtVQ35*		
*NtVQ41/NtVQ24*		
*NtVQ43/NtVQ44*		
*NtVQ44/NtVQ43*		
*NtVQ45/NtVQ49*		
*NtVQ46/NtVQ58*		
*NtVQ48/NtVQ50*		

**Table 2:** *Ka*, *Ks*, and *Ka/Ks* values calculated for paralogous *NtVQ* gene pairs.

Gene 1	Gene 2	*Ka*	*Ks*	*Ka/Ks*
*NtVQ10*	*NtVQ9*	0.0236959	0.0854132	0.277427
*NtVQ11*	*NtVQ12*	0.0336896	0.142241	0.236849
*NtVQ15*	*NtVQ16*	0.0247142	0.119972	0.206
*NtVQ17*	*NtVQ6*	0.0237371	0.0870851	0.272573
*NtVQ18*	*NtVQ19*	0.0288426	0.10777	0.267632
*NtVQ20*	*NtVQ23*	0.0699917	0.209588	0.333949
*NtVQ21*	*NtVQ22*	0.0353919	0.108026	0.327624
*NtVQ24*	*NtVQ41*	0.0322853	0.0903173	0.357465
*NtVQ25*	*NtVQ26*	0.00768491	0.128751	0.059688
*NtVQ27*	*NtVQ28*	0.0129349	0.0794967	0.162709
*NtVQ29*	*NtVQ36*	0.0660472	0.17302	0.381732
*NtVQ30*	*NtVQ31*	0.0151727	0.157758	0.096177
*NtVQ33*	*NtVQ34*	0.0446333	0.203136	0.219722
*NtVQ37*	*NtVQ38*	0.0175787	0.061387	0.286358
*NtVQ43*	*NtVQ44*	0.0217306	0.0879767	0.247004
*NtVQ45*	*NtVQ49*	0.0300847	0.15376	0.19566
*NtVQ48*	*NtVQ50*	0.0729002	0.32705	0.222902
*NtVQ48*	*NtVQ51*	0.0709646	0.408619	0.173669
*NtVQ48*	*NtVQ52*	0.0681005	0.386549	0.176176
*NtVQ48*	*NtVQ56*	0.079978	0.378101	0.211525
*NtVQ48*	*NtVQ57*	0.0695372	0.254073	0.273689
*NtVQ4*	*NtVQ5*	0.0233695	0.132132	0.176864
*NtVQ50*	*NtVQ51*	0.105789	0.36515	0.289714
*NtVQ50*	*NtVQ52*	0.100707	0.571344	0.176263
*NtVQ50*	*NtVQ56*	0.10983	0.306966	0.357792
*NtVQ50*	*NtVQ57*	0.104101	0.370383	0.281064
*NtVQ51*	*NtVQ52*	0.041644	0.142729	0.29177
*NtVQ51*	*NtVQ56*	0.119672	0.258776	0.462456
*NtVQ51*	*NtVQ57*	0.13037	0.264411	0.493056
*NtVQ52*	*NtVQ56*	0.0932197	0.292244	0.318979
*NtVQ52*	*NtVQ57*	0.0769438	0.236928	0.324756
*NtVQ53*	*NtVQ55*	0.025791	0.099437	0.25937
*NtVQ56*	*NtVQ57*	0.0442237	0.206447	0.214214
*NtVQ5*	*NtVQ4*	0.0233695	0.132132	0.176864

Note: The redundant pairs and the pairs with *p*-value > 0.05 are not listed this table.

## Supplementary Materials

The following are available online at https://www.mdpi.com/2073-4425/11/3/284/s1, Figure S1: Structural analysis of *NtVQ* genes. The blue arrows represent the exons, the green rectangle represents the introns and the black solid lines represent the upstream and downstream of the gene; Figure S2: Heat map showing significantly modulated *NtVQ* gene expression patterns in tobacco under phytohormone treatment. *NtVQ* gene expression levels were measured in two-week-old seedling under 2 mM SA, 100 μM JA, 1 mM ET or 100 μM ABA treatments post 0 h, 3 h and 24 h, respectively, compared to the CK plants. The relative expression value of each gene is colored based on the scale bar. Color changes from green to red in bar represent expression level changes from −3 to 3; Figure S3: Expression analysis of *NtVQ* genes in response to cold treatment. Two-week-old seedlings were treated at 4 °C for 0, 3 and 24 h before the whole plants were harvested. The relative expression value of each *NtVQ* gene was normalized to *NtEF1a* with CK as calibrator. CK, control group grown constantly at 25 °C. * indicates the level of significance relative to the CK value (* *p* < 0.05, ** *p* < 0.01); Figure S4: Expression analysis of *NtVQ* genes in response to heat treatment. Two-week-old seedlings were treated at 35 °C for 0, 3 and 24 h before the whole plants were harvested. The relative expression value of each *NtVQ* gene was normalized to *NtEF1a* with CK as calibrator. CK, control group grown constantly at 25 °C. * indicates the level of significance relative to the CK value (* *p* < 0.05, ** *p* < 0.01); Table S1: Oligonucleotide primers used in qRT-PCR analysis of *NtVQ* genes; Table S2: Analysis of *NtVQ* gene origin in progenitor species of cultivated tobacco; Table S3: Biochemical properties of the various NtVQ domain proteins in tobacco (*Nicotiana tabacum* L.); Table S4: The nucleotide coding sequences of various *NtVQ* genes and predicted amino acid sequences of their encoded proteins; Table S5: Expression analysis of *NtVQ* genes in response to different phytohormonal treatments; Table S6: Predicted transcription factor binding sites in the 5′-flanking regions of the *NtVQ* genes.

## References

[1] Oksman-Caldentey K.M. (2007). Tropane and nicotine alkaloid biosynthesis-novel approaches towards biotechnological production of plant-derived pharmaceuticals. Curr. Pharm. Biotechno..

[2] Rushton P.J., Bokowiec M.T., Han S.C., Zhang H.B., Brannock J.F., Chen X.F., Laudeman T.W., Timko M.P. (2008). Tobacco transcription factors: Novel insights into transcriptional regulation in the Solanaceae. Plant Physiol..

[3] Sierro N., Battey J.N.D., Ouadi S., Bakaher N., Bovet L., Willig A., Goepfert S., Peitsch M.C., Ivanov N.V. (2014). The tobacco genome sequence and its comparison with those of tomato and potato. Nat. Commun..

[4] Jones J.D.G., Dangl J.L. (2006). The plant immune system. Nature.

[5] Buscaill P., Rivas S. (2014). Transcriptional control of plant defence responses. Curr. Opin. Plant Biol..

[6] Colebrook E.H., Thomas S.G., Phillips A.L., Hedden P. (2014). The role of gibberellin signalling in plant responses to abiotic stress. J. Exp. Biol..

[7] Tsuda K., Somssich I.E. (2015). Transcriptional networks in plant immunity. New Phytol..

[8] Birkenbihl R.P., Liu S., Somssich I.E. (2017). Transcriptional events defining plant immune responses. Curr. Opin. Plant Biol..

[9] Van Ruyskensvelde V., Van Breusegem F., Van der Kelen K. (2018). Post-transcriptional regulation of the oxidative stress response in plants. Free Radic. Biol. Med..

[10] Jing Y.J., Lin R.C. (2015). The VQ motif-containing protein family of plant-specific transcriptional regulators. Plant Physiol..

[11] Cheng Y., Zhou Y., Yang Y., Chi Y.J., Zhou J., Chen J.Y., Wang F., Fan B.F., Shi K., Zhou Y.H. (2012). Structural and functional analysis of VQ motif-containing proteins in Arabidopsis as interacting proteins of WRKY transcription factors. Plant Physiol..

[12] Kim D.Y., Kwon S.I., Choi C., Lee H., Ahn L., Park S.R., Bae S.C., Lee S.C., Hwang D.J. (2013). Expression analysis of rice VQ genes in response to biotic and abiotic stresses. Gene.

[13] Wang M., Vannozzi A., Wang G., Zhong V., Corso M., Cavallini E., Cheng Z.M. (2015). A comprehensive survey of the grapevine VQ gene family and its transcriptional correlation with WRKY proteins. Front. Plant Sci..

[14] Zhang G.Y., Wang F.D., Li J.J., Ding Q., Zhang Y.H., Li H.Y., Zhang J.N., Gao J.W. (2015). Genome-wide identification and analysis of the VQ motif-containing protein family in Chinese cabbage (*Brassica rapa* L. ssp. Pekinensis). Int. J. Mol. Sci..

[15] Chu W.Y., Liu B., Wang Y.J., Pan F., Chen Z., Yan H.W., Xiang Y. (2016). Genome-wide analysis of poplar VQ gene family and expression profiling under PEG, NaCl, and SA treatments. Tree Genet. Genomes.

[16] Song W.B., Zhao H.M., Zhang X.B., Lei L., Lai J.S. (2016). Genome-wide identification of VQ motif-containing proteins and their expression profiles under abiotic stresses in maize. Front. Plant Sci..

[17] Zhou Y., Yang Y., Zhou X.J., Chi Y.J., Fan B.F., Chen Z.X. (2016). Structural and functional characterization of the VQ protein family and VQ protein variants from soybean. Sci. Rep..

[18] Wang Y.J., Liu H.L., Zhu D.Y., Gao Y.M., Yan H.W., Xiang Y. (2017). Genome-wide analysis of VQ motif-containing proteins in Moso bamboo (*Phyllostachys edulis*). Planta.

[19] Cao Y.P., Meng D.D., Abdullah M., Jin Q., Lin Y., Cai Y.P. (2018). Genome wide identification, evolutionary, and expression analysis of VQ genes from two *Pyrus* species. Genes.

[20] Dong Q.L., Zhao S., Duan D.Y., Tian Y., Wang Y.P., Mao K., Zhou Z.S., Ma F.W. (2018). Structural and functional analyses of genes encoding VQ proteins in apple. Plant Sci..

[21] Zhong Y., Guo C., Chu J.J., Liu H., Cheng Z.M. (2018). Microevolution of the VQ gene family in six species of *Fragaria*. Genome.

[22] Wang Y., Jiang Z., Li Z., Zhao Y., Tan W., Liu Z., Cui S., Yu X., Ma J., Wang G. (2019). Genome-wide identification and expression analysis of the VQ gene family in soybean (*Glycine max*). Peerj.

[23] Guo J., Chen J., Yang J., Yu Y., Yang Y., Wang W. (2018). Identification, characterization and expression analysis of the VQ motif-containing gene family in tea plant (*Camellia sinensis*). BMC Genom..

[24] Ding H., Yuan G., Mo S., Qian Y., Wu Y., Chen Q., Xu X., Wu X., Ge C. (2019). Genome-wide analysis of the plant-specific VQ motif-containing proteins in tomato (*Solanum lycopersicum*) and characterization of SlVQ6 in thermotolerance. Plant Physiol. Biochem..

[25] Ye Y.-J., Xiao Y.-Y., Han Y.-C., Shan W., Fan Z.-Q., Xu Q.-G., Kuang J.-F., Lu W.-J., Lakshmanan P., Chen J.-Y. (2016). Banana fruit VQ motif-containing protein5 represses cold-responsive transcription factor MaWRKY26 involved in the regulation of JA biosynthetic genes. Sci. Rep..

[26] Jiang S.Y., Sevugan M., Ramachandran S. (2018). Valine-glutamine (VQ) motif coding genes are ancient and non-plant-specific with comprehensive expression regulation by various biotic and abiotic stresses. BMC Genom..

[27] Pecher P., Eschen-Lippold L., Herklotz S., Kuhle K., Naumann K., Bethke G., Uhrig J., Weyhe M., Scheel D., Lee J. (2014). The *Arabidopsis thaliana* mitogen-activated protein kinases MPK3 and MPK6 target a subclass of “VQ-motif’-containing proteins to regulate immune responses. New Phytol..

[28] Hu Y.R., Chen L.G., Wang H.P., Zhang L.P., Wang F., Yu D.Q. (2013). Arabidopsis transcription factor WRKY8 functions antagonistically with its interacting partner VQ9 to modulate salinity stress tolerance. Plant J..

[29] Wang A.H., Garcia D., Zhang H.Y., Feng K., Chaudhury A., Berger F., Peacock W.J., Dennis E.S., Luo M. (2010). The VQ motif protein IKU1 regulates endosperm growth and seed size in Arabidopsis. Plant J..

[30] Perruc E., Charpenteau M., Ramirez B.C., Jauneau A., Galaud J.P., Ranjeva R., Ranty B. (2004). A novel calmodulin-binding protein functions as a negative regulator of osmotic stress tolerance in *Arabidopsis thaliana* seedlings. Plant J..

[31] Lei R., Li X., Ma Z., Lv Y., Hu Y., Yu D. (2017). Arabidopsis WRKY2 and WRKY34 transcription factors interact with VQ20 protein to modulate pollen development and function. Plant J..

[32] Andreasson E., Jenkins T., Brodersen P., Thorgrimsen S., Petersen N.H.T., Zhu S.J., Qiu J.L., Micheelsen P., Rocher A., Petersen M. (2005). The MAP kinase substrate MKS1 is a regulator of plant defense responses. EMBO J..

[33] Qiu J.L., Fiil B.K., Petersen K., Nielsen H.B., Botanga C.J., Thorgrimsen S., Palma K., Suarez-Rodriguez M.C., Sandbech-Clausen S., Lichota J. (2008). Arabidopsis MAP kinase 4 regulates gene expression through transcription factor release in the nucleus. EMBO J..

[34] Gargul J.M., Mibus H., Serek M. (2015). Manipulation of *MKS1* gene expression affects *Kalanchoë blossfeldiana* and *Petunia hybrida* phenotypes. Plant Biotechnol. J..

[35] Hu P., Zhou W., Cheng Z.W., Fan M., Wang L., Xie D.X. (2013). JAV1 controls jasmonate-regulated plant defense. Mol. Cell.

[36] Ali M.R.M., Uemura T., Ramadan A., Adachi K., Nemoto K., Nozawa A., Hoshino R., Abe H., Sawasaki T., Arimura G.I. (2019). The ring-type E3 ubiquitin ligase JUL1 targets the VQ-motif protein JAV1 to coordinate jasmonate signaling. Plant Physiol..

[37] Lai Z.B., Li Y., Wang F., Cheng Y., Fan B.F., Yu J.Q., Chen Z.X. (2011). Arabidopsis sigma factor binding proteins are activators of the WRKY33 transcription factor in plant defense. Plant Cell.

[38] Pan J., Wang H., Hu Y., Yu D. (2018). Arabidopsis VQ18 and VQ26 proteins interact with ABI5 transcription factor to negatively modulate ABA response during seed germination. Plant J..

[39] Li Y.L., Jing Y.J., Li J.J., Xu G., Lin R.C. (2014). Arabidopsis VQ MOTIF-CONTAINING PROTEIN29 represses seedling deetiolation by interacting with PHYTOCHROME-INTERACTING FACTOR1. Plant Physiol..

[40] Murashige T., Skoog F. (1962). A Revised medium for rapid growth and bio assays with tobacco tissue cultures. Physiol. Plantarum..

[41] Jones D.T., Taylor W.R., Thornton J.M. (1992). The rapid generation of mutation data matrices from protein sequences. Comput. Appl. Biosci..

[42] Kumar S., Stecher G., Tamura K. (2016). MEGA7: Molecular evolutionary genetics analysis version 7.0 for bigger datasets. Mol. Biol. Evol..

[43] Bailey T.L., Williams N., Misleh C., Li W.W. (2006). MEME: Discovering and analyzing DNA and protein sequence motifs. Nucleic Acids Res..

[44] Bailey T.L., Boden M., Buske F.A., Frith M., Grant C.E., Clementi L., Ren J.Y., Li W.W., Noble W.S. (2009). MEME SUITE: Tools for motif discovery and searching. Nucleic Acids Res..

[45] Wang D., Zhang Y., Zhang Z., Zhu J., Yu J. (2010). KaKs_Calculator 2.0: A Toolkit Incorporating γ-Series Methods and Sliding Window Strategies. Genom. Proteom. Bioinform..

[46] Gaut B.S. (1998). Molecular clocks and nucleotide substitution rates in higher plants. Evol. Biol..

[47] Kim M.Y., Kang Y.J., Lee T., Lee S.H. (2013). Divergence of flowering-related genes in three legume species. Plant Genome-Us.

[48] Lescot M., Dehais P., Thijs G., Marchal K., Moreau Y., Van de Peer Y., Rouze P., Rombauts S. (2002). PlantCARE, a database of plant *cis*-acting regulatory elements and a portal to tools for in silico analysis of promoter sequences. Nucleic Acids Res..

[49] Schmidt G.W., Delaney S.K. (2010). Stable internal reference genes for normalization of real-time RT-PCR in tobacco (*Nicotiana tabacum*) during development and abiotic stress. Mol. Genet. Genom..

[50] Kaul S., Koo H.L., Jenkins J., Rizzo M., Rooney T., Tallon L.J., Feldblyum T., Nierman W., Benito M.I., Lin X.Y. (2000). Analysis of the genome sequence of the flowering plant *Arabidopsis thaliana*. Nature.

[51] Yu J., Ni P.X., Wong G.K.S. (2006). Comparing the whole-genome-shotgun and map-based sequences of the rice genome. Trends Plant Sci..

[52] Daccord N., Celton J.M., Linsmith G., Becker C., Choisne N., Schijlen E., van de Geest H., Bianco L., Micheletti D., Velasco R. (2017). High-quality de novo assembly of the apple genome and methylome dynamics of early fruit development. Nat. Genet..

[53] Leitch I.J., Hanson L., Lim K.Y., Kovarik A., Chase M.W., Clarkson J.J., Leitch A.R. (2008). The ups and downs of genome size evolution in polyploid species of *Nicotiana* (*Solanaceae*). Ann. Bot..

[54] Renny-Byfield S., Chester M., Kovarik A., Le Comber S.C., Grandbastien M.A., Deloger M., Nichols R.A., Macas J., Novak P., Chase M.W. (2011). Next generation sequencing reveals genome downsizing in allotetraploid *Nicotiana tabacum*, predominantly through the elimination of paternally derived repetitive DNAs. Mol. Biol. Evol..

[55] Li N., Li X.H., Xiao J.H., Wang S.P. (2014). Comprehensive analysis of VQ motif-containing gene expression in rice defense responses to three pathogens. Plant Cell Rep..

[56] Garrido-Gala J., Higuera J.J., Munoz-Blanco J., Amil-Ruiz F., Caballero J.L. (2019). The VQ motif-containing proteins in the diploid and octoploid strawberry. Sci. Rep..

[57] DebRoy S., Thilmony R., Kwack Y.B., Nomura K., He S.Y. (2004). A family of conserved bacterial effectors inhibits salicylic acid-mediated basal immunity and promotes disease necrosis in plants. Proc. Natl. Acad. Sci. USA.

[58] Ohtsubo N., Mitsuhara I., Koga M., Seo S., Ohashi Y. (1999). Ethylene promotes the necrotic lesion formation and basic PR gene expression in TMV-infected tobacco. Plant Cell Physiol..

[59] Song S.S., Qi T.C., Wasternack C., Xie D.X. (2014). Jasmonate signaling and crosstalk with gibberellin and ethylene. Curr. Opin. Plant Biol..

[60] Yan H.F., Wang Y.J., Hu B., Qiu Z.F., Zeng B.S., Fan C.J. (2019). Genome-wide characterization, evolution, and expression profiling of *VQ* gene family in response to phytohormone treatments and abiotic stress in *Eucalyptus grandis*. Int. J. Mol. Sci..

[61] Dong J.X., Chen C.H., Chen Z.X. (2003). Expression profiles of the *Arabidopsis* WRKY gene superfamily during plant defense response. Plant Mol. Biol..

[62] Chi Y.J., Yang Y., Zhou Y., Zhou J., Fan B.F., Yu J.Q., Chen Z.X. (2013). Protein-protein interactions in the regulation of WRKY transcription factors. Mol. Plant.

[63] Weyhe M., Eschen-Lippold L., Pecher P., Scheel D., Lee J. (2014). Menage a trois: The complex relationships between mitogen-activated protein kinases, WRKY transcription factors, and VQ-motif-containing proteins. Plant Signal. Behav..

[64] Ross C.A., Liu Y., Shen Q.X.J. (2007). The *WRKY* gene family in rice (*Oryza sativa*). J. Integr. Plant Biol..

[65] Rushton P.J., Somssich I.E., Ringler P., Shen Q.X.J. (2010). WRKY transcription factors. Trends Plant Sci..

[66] Yan C., Fan M., Yang M., Zhao J.P., Zhang W.H., Su Y., Xiao L.T., Deng H.T., Xie D.X. (2018). Injury activates Ca_2+_/calmodulin-dependent phosphorylation of JAV1-JAZ8-WRKY51 complex for jasmonate biosynthesis. Mol. Cell.

