# Supplementary figures and images for: Genome-Wide Identification of the VQ Protein Gene Family of Tobacco (Nicotiana tabacum L.) and Analysis of Its Expression in Response to Phytohormones and Abiotic and Biotic Stresses
Source: Genes (Basel). 2020 Mar 7;11(3):284. doi: 10.3390/genes11030284 (PMC7140788; doi:10.3390/genes11030284)

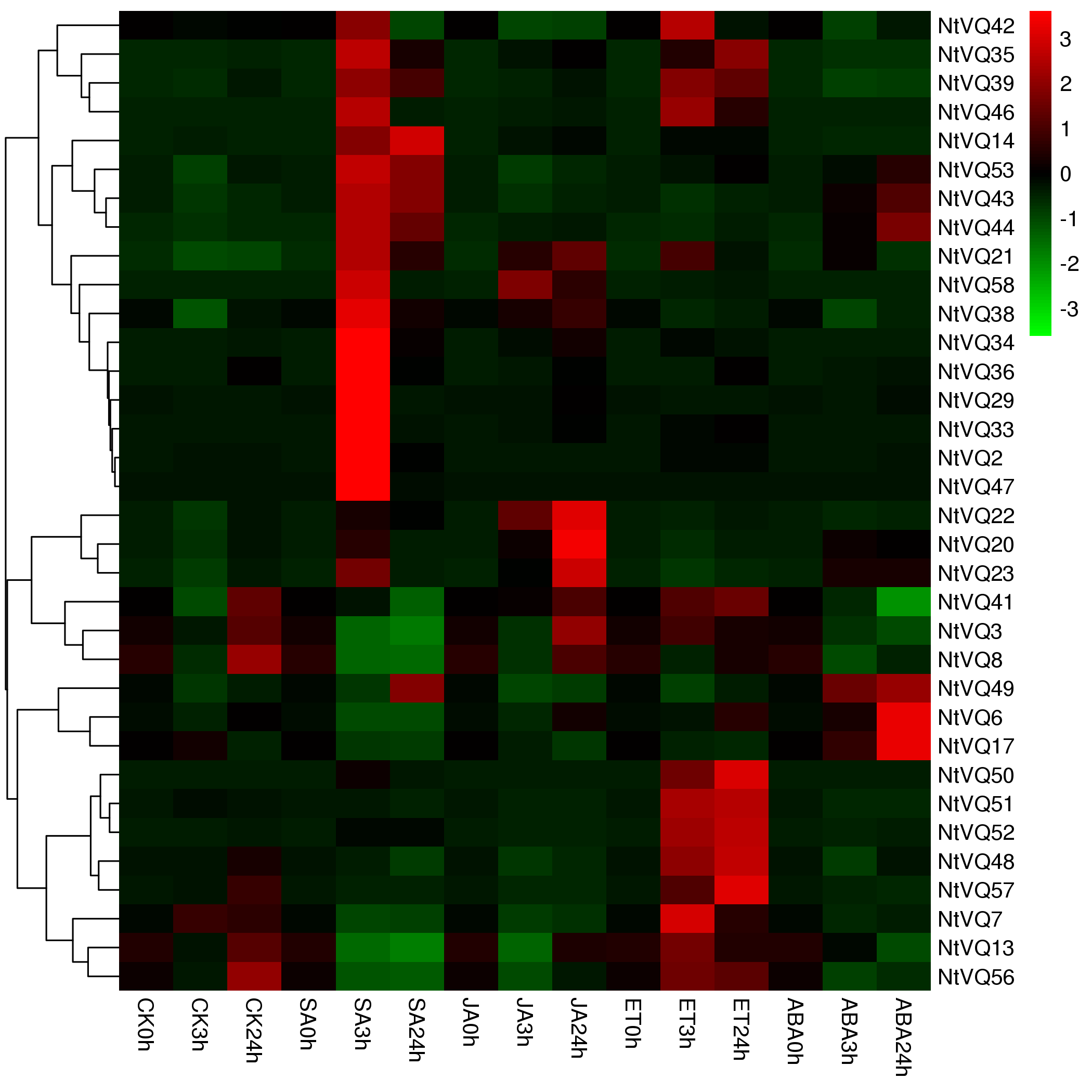

Supplement: Supplementary file 1 [file genes-11-00284-s001.zip › Supplementary Figure S2.png]

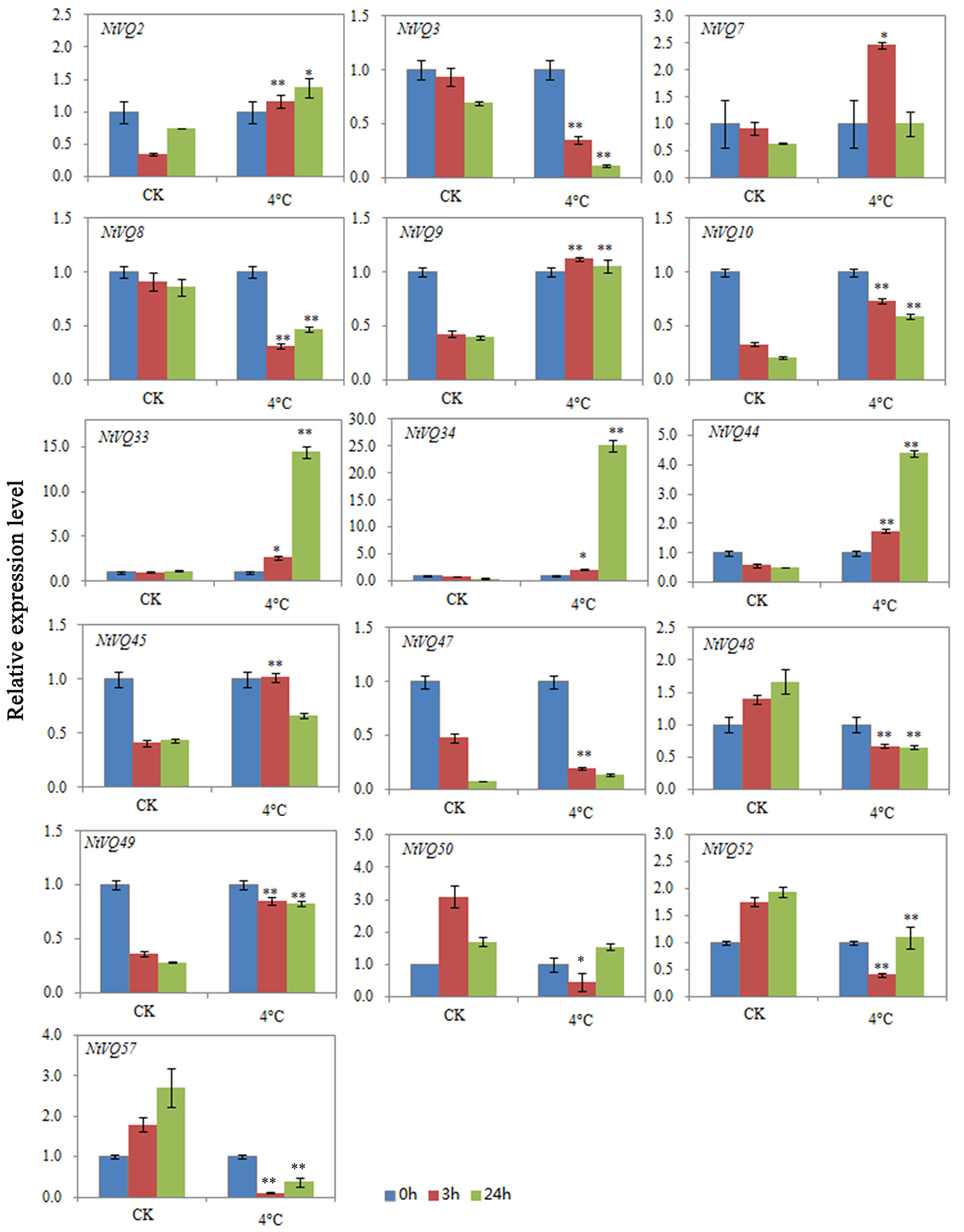

Supplement: Supplementary file 1 [file genes-11-00284-s001.zip › Supplementary Figure S3.jpg]

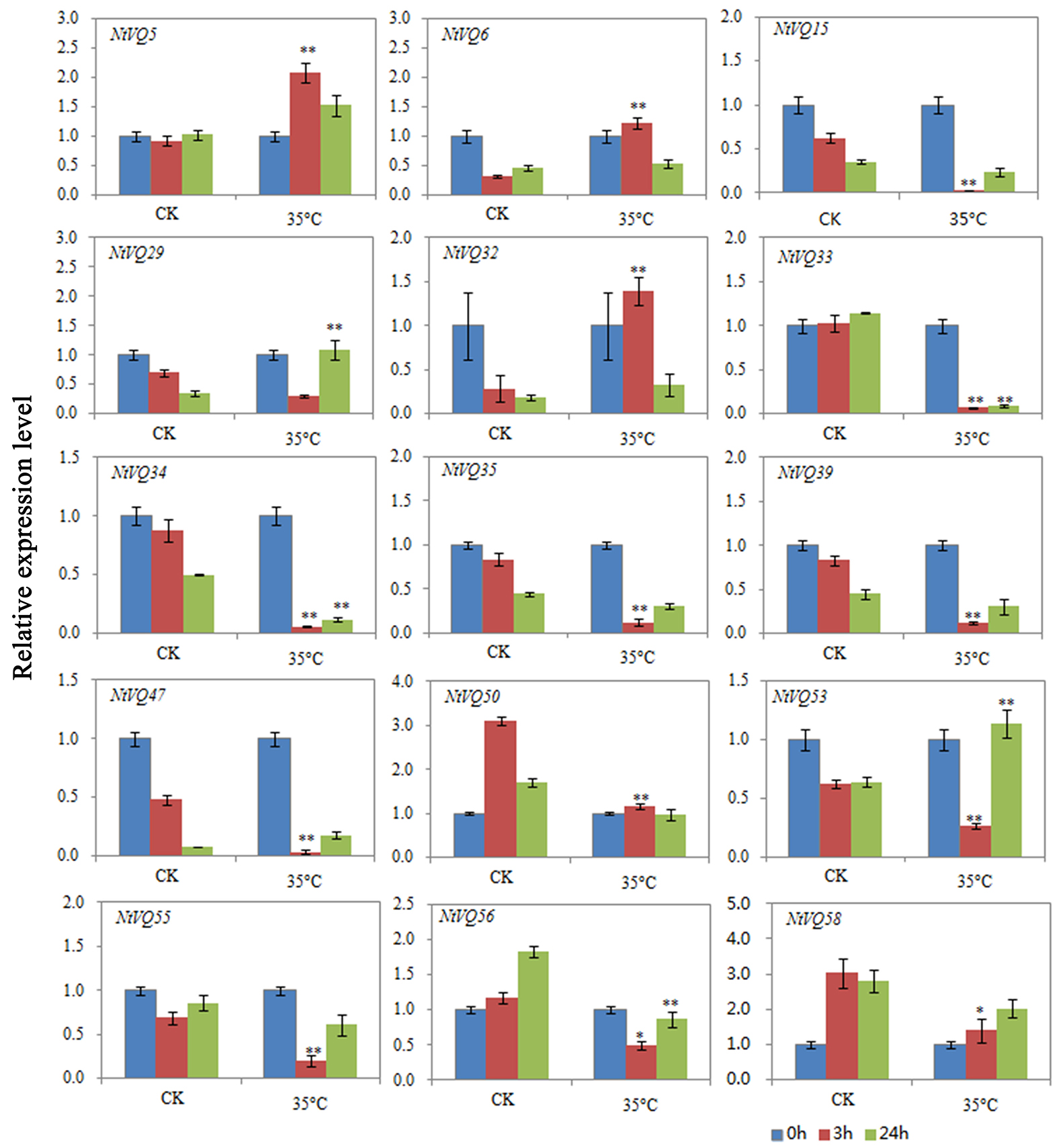

Supplement: Supplementary file 1 [file genes-11-00284-s001.zip › Supplementary Figure S4.jpg]

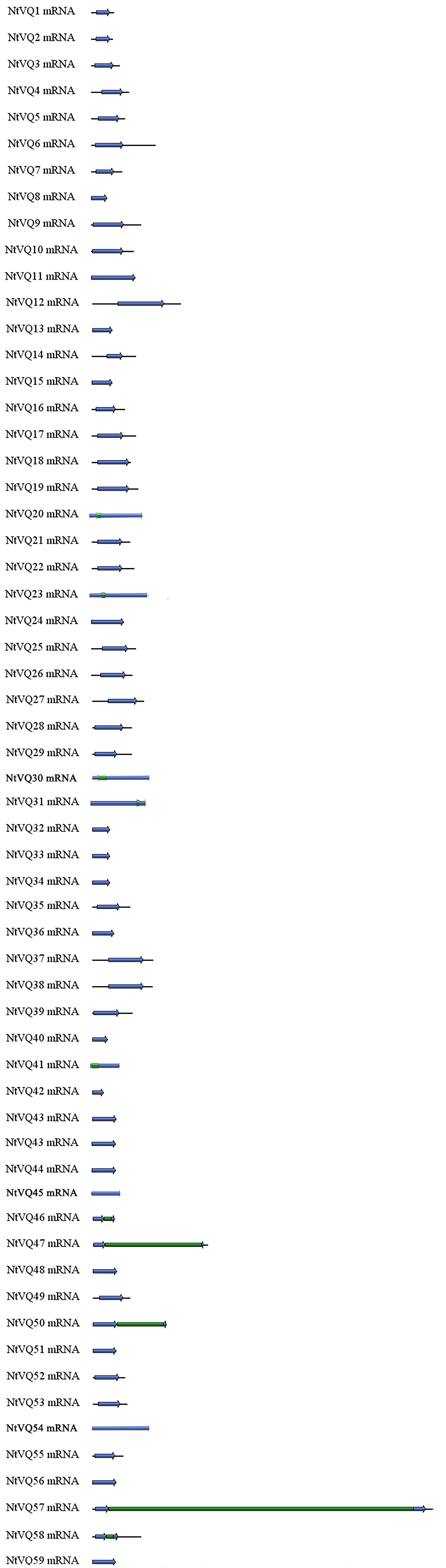

Supplement: Supplementary file 1 [file genes-11-00284-s001.zip › Supplementary Figure S1.jpg]
